# Ultrasound-Based Fluid-Structure Interaction Modeling of Abdominal Aortic Aneurysms Incorporating Pre-stress

**DOI:** 10.3389/fphys.2021.717593

**Published:** 2021-08-13

**Authors:** Judith H. C. Fonken, Esther J. Maas, Arjet H. M. Nievergeld, Marc R. H. M. van Sambeek, Frans N. van de Vosse, Richard G. P. Lopata

**Affiliations:** ^1^Photoacoustics & Ultrasound Laboratory Eindhoven (PULS/e), Department of Biomechanical Engineering, Eindhoven University of Technology, Eindhoven, Netherlands; ^2^Department of Vascular Surgery, Catharina Hospital Eindhoven, Eindhoven, Netherlands; ^3^Cardiovascular Biomechanics, Department of Biomechanical Engineering, Eindhoven University of Technology, Eindhoven, Netherlands

**Keywords:** abdominal aortic aneurysms, patient-specific, fluid-structure interaction modeling, rupture risk, pre-stress estimation, wall mechanics, hemodynamics

## Abstract

Currently, the prediction of rupture risk in abdominal aortic aneurysms (AAAs) solely relies on maximum diameter. However, wall mechanics and hemodynamics have shown to provide better risk indicators. Patient-specific fluid-structure interaction (FSI) simulations based on a non-invasive image modality are required to establish a patient-specific risk indicator. In this study, a robust framework to execute FSI simulations based on time-resolved three-dimensional ultrasound (3D+t US) data was obtained and employed on a data set of 30 AAA patients. Furthermore, the effect of including a pre-stress estimation (PSE) to obtain the stresses present in the measured geometry was evaluated. The established workflow uses the patient-specific 3D+t US-based segmentation and brachial blood pressure as input to generate meshes and boundary conditions for the FSI simulations. The 3D+t US-based FSI framework was successfully employed on an extensive set of AAA patient data. Omitting the pre-stress results in increased displacements, decreased wall stresses, and deviating time-averaged wall shear stress and oscillatory shear index patterns. These results underline the importance of incorporating pre-stress in FSI simulations. After validation, the presented framework provides an important tool for personalized modeling and longitudinal studies on AAA growth and rupture risk.

## 1. Introduction

An abdominal aortic aneurysm (AAA) is a pathological dilation of the aorta beyond 50% of the normal vessel diameter. If left untreated, the AAA can expand until rupture occurs, which is accompanied by an overall mortality of 80% (Rissland et al., 2008; Piechota-Polanczyk et al., 2015; Kontopodis et al., 2018; Salman et al., 2019). Surgical repair of AAAs can be performed to prevent rupture, but is not without risks either (Kontopodis et al., 2018). Therefore, after a patient is diagnosed with an AAA, the patient's risk of rupture is assessed and monitored over time. Current clinical estimates of rupture risk are based on the maximum diameter and growth of the aneurysm. Surgical aneurysm repair is recommended when the maximum diameter exceeds 5.0 cm (women) or 5.5 cm (men), or when the growth rate is over 1 cm/year (Chaikof et al., 2018; Kontopodis et al., 2018; Salman et al., 2019). The adoption of this measure for rupture risk was based on randomized clinical trials (Collin, 1999; Lederle et al., 2002). However, a retrospective review reported that ~40% of AAAs between 7 and 10 cm did not rupture, whereas 13% of AAAs with a maximum diameter below 5 cm did rupture (Chaikof et al., 2018). These findings suggest that maximum diameter alone is an inadequate indicator for rupture risk and that another approach is needed to predict the rupture risk (Rissland et al., 2008; van Disseldorp et al., 2016; Chaikof et al., 2018; Salman et al., 2019).

From a mechanical point of view, AAA rupture occurs when the stresses acting on the aneurysm wall exceed the strength of the aortic wall (Rissland et al., 2008; van Disseldorp et al., 2016; Chaikof et al., 2018; Salman et al., 2019). Since multiple studies have demonstrated that stresses acting on the wall are highly dependent on AAA geometry, a patient-specific risk assessment is required (Chaikof et al., 2018; Salman et al., 2019). Computational solid stress (CSS) models using patient-specific geometries have been employed in a variety of previous studies and were successful in predicting the wall stresses and potential rupture sites (Fillinger et al., 2003; Vorp, 2007; Xenos and Bluestein, 2011; van Disseldorp et al., 2016; Chaikof et al., 2018).

Besides CSS analyses, various computational fluid dynamic (CFD) analyses have been executed to study AAA development and growth (Biasetti et al., 2010; Les et al., 2010; Xenos and Bluestein, 2011; Boyd et al., 2016; Zambrano et al., 2016; Salman et al., 2019). It is believed that low, disturbed wall shear stress (WSS) in the AAA activates inflammatory markers, which might lead to degeneration and weakening of the vessel wall and formation of intraluminal thrombus (ILT) (Xenos and Bluestein, 2011; Tanweer et al., 2014; Zambrano et al., 2016; Salman et al., 2019). The influence of ILT on AAA progression and rupture is still ambiguous. On the one hand, ILT potentially affects the AAA wall strength negatively by obstructing the transport of solutes and disrupting the direct interaction between hemodynamic forces and the vessel wall (Vorp et al., 2001; Zambrano et al., 2016). On the other hand, studies suggested that the ILT could prevent AAA rupture by lowering the wall stress (Vorp, 2007; Speelman et al., 2010; Xenos and Bluestein, 2011; Zambrano et al., 2016).

Research on AAA growth and rupture risk requires a longitudinal study on a large set of patients. Furthermore, fluid-structure interaction (FSI) models need to be employed to analyze the interaction between hemodynamics and wall mechanics (Lin et al., 2017; Salman et al., 2019) and to investigate ILT formation. In CFD analyses, the wall is assumed to be rigid, which leads to an underestimation of vortex development and overestimation of WSS (Lin et al., 2017). In CSS simulations, an uniform pressure is applied to the AAA wall, which can lead to an underestimation of wall stresses up to 10% (Lin et al., 2017).

Previous studies employing FSI simulations either used idealized AAA geometries (Scotti et al., 2005, 2008; Lin et al., 2017) or a small set of patient-specific geometries acquired using computed tomography (CT) (Di Martino et al., 2001; Wolters et al., 2005; Scotti and Finol, 2007; Xenos and Bluestein, 2011). Due to the use of radiation and contrast agents, CT is unsuitable for frequent use, and thus large longitudinal studies (Salman et al., 2019). In contrast, time-resolved 3-dimensional ultrasound (3D+t US) is safe, fast, affordable, and contains geometric information during the full cardiac cycle. Furthermore, (2D) ultrasound is already used in the current clinical workflow of AAA surveillance. However, 3D+t US is not frequently used to acquire the patient-specific geometries for finite element models due to its limitations in contrast and field-of-view with respect to CT. Recent improvements in 3D+t US segmentation methods allow for the use of 3D+t US data in CSS models, including the use of the geometric information for simultaneous estimation of patient-specific wall stresses and stiffness, as demonstrated by van Disseldorp et al. (2018).

In addition, previous FSI studies have neglected the pre-stress present in the measured AAA geometry due to the physiological pressure load that is present during image acquisition. Assuming the measured AAA geometry to be unloaded and unstressed has shown to influence the wall mechanics in CSS simulations (de Putter et al., 2007; Bols et al., 2013). Since the wall mechanics influence the hemodynamics and vice versa, incorporating the pre-stress in FSI models is deemed necessary. Hence, in this study, the effect of incorporating the pre-stress was evaluated for each patient by executing an additional FSI simulation without pre-stress estimation.

In this study, a robust, automated framework to execute FSI simulations of AAAs based on 3D+t US data was developed and employed to simulate the wall mechanics and hemodynamics of 30 AAA patients. Furthermore, the effect of incorporating the pre-stress was evaluated for each patient by executing an additional FSI simulation without pre-stress estimation.

## 2. Materials and Methods

In a collaborative study, 3D+t US acquisitions of 30 AAA patients were acquired in the Catharina Hospital in Eindhoven. The study was approved by the local ethics committee and all patients gave their written informed consent. The patients were divided in three equal groups based on the measured maximum diameter: small (S, ≤39 mm), moderate (M, 40–49 mm) and large (L, ≥50 mm) (van Disseldorp et al., 2018). The patient-specific brachial BP was measured in supine position immediately after image acquisition using a pressure cuff. The maximum diameter, length of the AAA geometry and the brachial BP for all patients are summarized in Table 1. van't Veer et al. (2008) have demonstrated that brachial cuff measurements overestimate the diastolic blood pressure (*P*_*dia*_) by 12% and underestimate the systolic blood pressure (*P*_*sys*_) by 5% in AAA patients. Therefore, the brachial BP was converted into the abdominal aortic (AA) BP using Equation (1).


(1) $$\begin{array}{l} P_{dia,AA}=0.88\cdot P_{dia,brachial}\\ P_{sys,AA}=1.05\cdot P_{sys,brachial} \end{array}$$

**Table 1:** Summary of the maximum diameter (D_max_), length of the AAA geometry (L_AAA_, elongation excluded) and brachial diastolic blood pressure (P_dia_), and systolic blood pressure (P_sys_) for all 30 patients and the (group) average (μ).

	**D_max_ (mm)**	**L_AAA_ (cm)**	**Brachial BP (mmHg)**			**D_max_ (mm)**	**L_AAA_ (cm)**	**Brachial BP (mmHg)**			**D_max_ (mm)**	**L_AAA_ (cm)**	**Brachial BP (mmHg)**	
			**P_dia_**	**P_sys_**				**P_dia_**	**P_sys_**				**P_dia_**	**P_sys_**
S1	27	6.6	80	141	M1	44	6.5	80	148	L1	50	5.9	73	138
S2	29	8.9	86	140	M2	44	6.6	99	179	L2	51	4.5	70	110
S3	30	6.9	83	122	M3	45	9.5	61	103	L3	51	9.2	91	137
S4	31	4.1	62	108	M4	45	7.1	78	136	L4	52	6.9	95	147
S5	33	5.3	82	129	M5	48	8.3	88	136	L5	53	8.9	87	147
S6	34	8.5	86	139	M6	48	7.9	86	145	L6	53	6.7	94	159
S7	35	7.7	74	125	M7	49	9.0	96	149	L7	54	8.4	92	142
S8	37	8.2	87	147	M8	49	6.6	67	122	L8	54	6.7	77	122
S9	37	7.2	82	133	M9	49	5.3	74	123	L9	55	9.7	81	143
S10	38	8.8	79	137	M10	49	7.8	83	132	L10	56	5.6	71	107
μS	33	7.2	80	132	μM	47	7.5	81	137	μL	53	7.2	83	135
μ	44	7.3	81	135										

*The patients were divided into three groups, based on the maximum diameter: small (S), moderate (M), and large (L)*.

**Figure 1:**
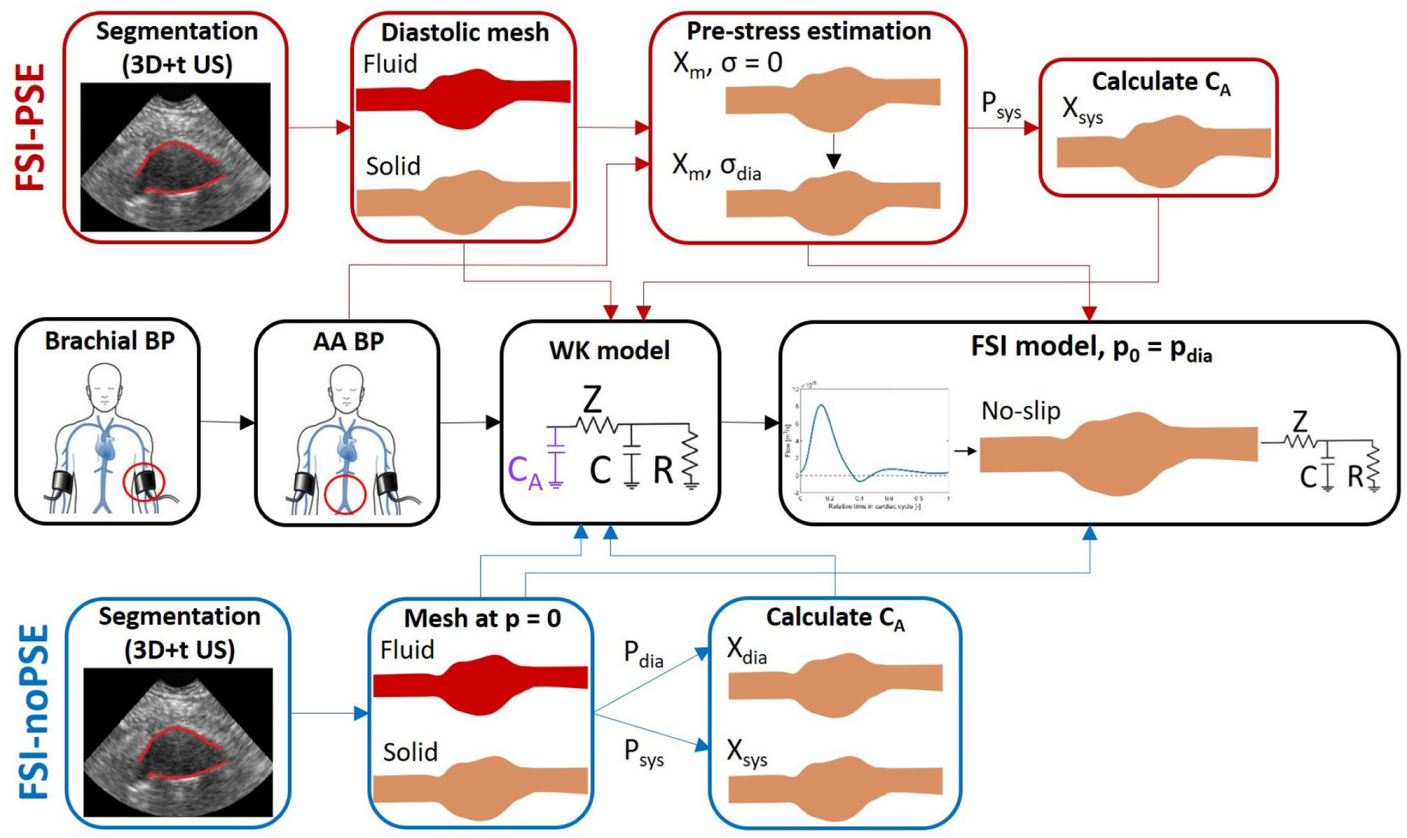
FSI simulation pipeline with PSE (FSI-PSE, red) and without PSE (PSI-noPSE, blue). Here, 3D+t US-based segmentations and brachial blood pressure (BP) are used as patient-specific input. Note that the black boxes are used in both FSI-PSE and FSI-noPSE pipelines.

A workflow was developed to execute FSI simulations of AAAs either with or without pre-stress estimation (PSE) (see Figure 1). For each workflow, the 3D+t, US-based segmentation and brachial blood pressure (BP) were used as inputs to create the fluid and structural meshes and boundary conditions for the FSI models, respectively. The complete workflow will be discussed in the subsequent sections.

### 2.1. Meshing

Using an in-house toolbox, based on the Star-Kalman method of Guerrero et al. (2007) and developed in MATLAB (R2020a, Mathworks Inc., Natick, MA, USA) (de Ruijter et al., 2020), the AAA geometry was automatically segmented from the 3D+t US acquisitions. For each frame, the phase in the heart cycle was determined by calculating the diameter change over time, and the segmentation at the diastolic frame was selected. To reduce the effects of the boundary conditions on the simulation results in the aneurysm region and avoid numerical instabilities (van Disseldorp et al., 2016; Zambrano et al., 2016), the segmentations were elongated by 5 cm in both proximal and distal direction using Bezier curves, resulting in a circular inlet and outlet with a fixed diameter of 2 cm. In proximal direction, the elongation resulted in a vessel parallel to the spine. In distal direction, the AAA geometry was elongated in the centerline direction (van Disseldorp et al., 2016). The segmentation was interpolated to obtain the desired element size of 0.8 mm and a lumen surface mesh was created by connecting the segmentation contours in quadrangular faces. The resulting lumen surface meshes for all the patients are shown in Supplementary Figure 1. This figure demonstrates the large variety in AAA geometries captured in this study.

**Figure 2:**
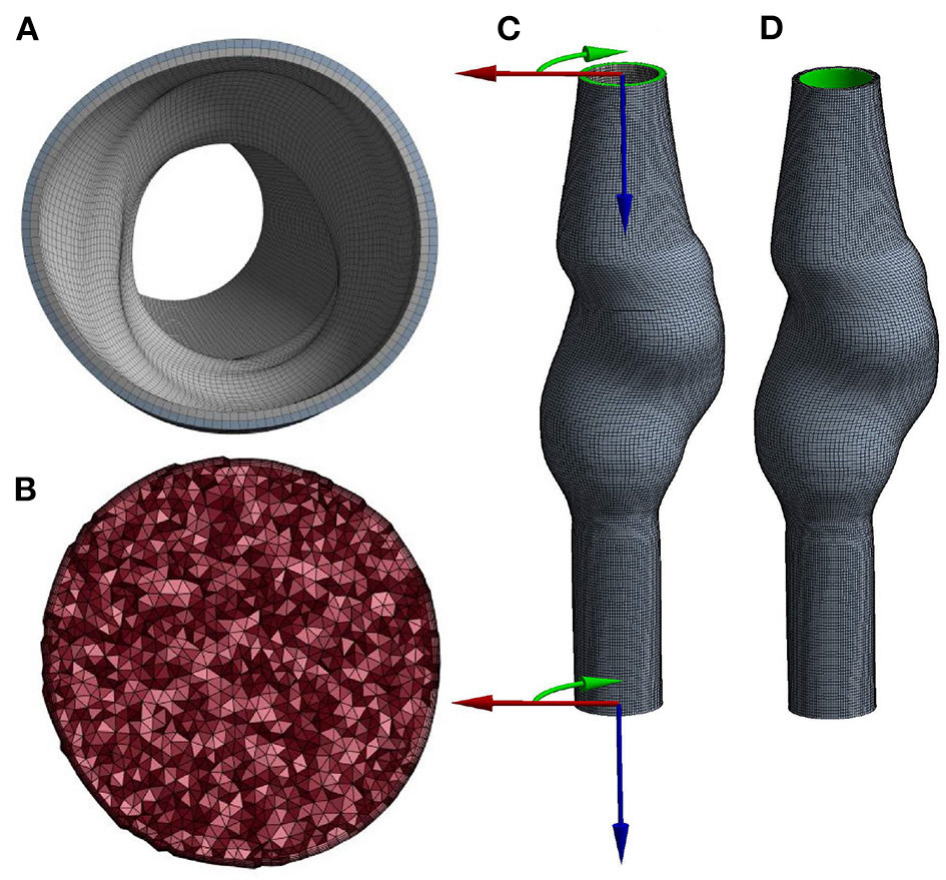
Cross-section of the **(A)** solid and **(B)** fluid volume mesh, and a visualization of **(C)** the boundary conditions at the inlet and outlet, using cylindrical coordinate systems and **(D)** the area (highlighted in green) to which pressure is applied.

The mesh for the AAA wall (solid domain) was obtained by extruding the lumen surface mesh and connecting the surface meshes using quadratic hexahedral elements. The wall thickness equals 2 mm and was equally divided over two layers of elements (Figure 2A).

For the fluid domain, the lumen surface mesh was triangulated, capped, and exported to Ansys Fluent (Ansys Inc., Canonsburg, PA, USA, version 2020R1) to subsequently create the lumen volume mesh. The boundary layer of the volume mesh consists of five layers of prism elements. The remaining volume was filled with tetrahedral elements with an element size of 1 mm (Figure 2B).

The optimal mesh sizes and number of wall layers were obtained by executing a mesh convergence study (Supplementary Material, section 2).

### 2.2. Structural Domain

The AAA wall was modeled as an isotropic, incompressible, Neo-Hookean material. The Neo-Hookean model relates the Cauchy stress (**σ**) and the left Cauchy Green deformation tensor (***B*** = ***F***·***F***^*c*^) according to Equation (2).


(2) $$\begin{array}{l} \sigma =-pI+G\left(B-I\right) \end{array}$$

With *G* the shear modulus of the material. This model is constitutively linear, yet geometrically non-linear. Although this model is not suitable to describe the typical non-linear properties of AAA tissue, from unloaded to fully loaded state (Holzapfel et al., 2012), it is sufficient for the purpose of this model, which is describing the material behavior of the wall within the physiological pressure range (van Disseldorp et al., 2016; Petterson et al., 2020). The shear modulus of the wall depends on the maximum diameter of the AAA, as quantified by van Disseldorp et al. (2018). Therefore, the shear modulus was set to 0.92, 1.02, and 1.36 MPa for the small, moderate, and large AAAs, respectively.

Local cylindrical coordinate systems (Figure 2C) were defined at the inlet and outlet of the mesh and used to constrain displacements in longitudinal and azimuthal directions. Therefore, the inlet and outlet nodes were only allowed to move in radial direction. The pressure was applied to the luminal surface of the wall (Figure 2D). The structural model was solved using Ansys Mechanical APDL (Ansys Inc., Canonsburg, PA, USA, version 2020R1).

The influence of incorporating the pre-stress in FSI models was evaluated by executing FSI simulations with and without incorporating a pre-stress estimation (PSE). In the workflow with PSE (FSI-PSE, Figure 1), the pre-stress was estimated using the Backward Incremental Method (BIM), introduced by de Putter et al. (2007). The advantage of the BIM compared to other pre-stress estimation methods is the direct estimation of the pre-stress in the measured geometry. In other approaches, such as the backward displacement method of Bols et al. (2013), the unloaded geometry first needs to be estimated, which imposes restrictions on the material model that is used.

In the BIM, the stress in the wall of the measured geometry is computed using an iterative approach, in which an incremental pressure is applied to the measured geometry using the stress state calculated in the previous iteration:


(3) $$\begin{array}{l} \text{Initialization}:{\hat{\Omega }}_{m}(\vec{x},\sigma )=\Omega_{m}({\vec{x}}_{m},0),p_{0}=0\\ \text{for }i=1:i_{max}\\ \;p_{i}=p_{m}\;sin(\frac{i\pi }{2i_{max}})\\ \;{\hat{\Omega }}_{m}({\vec{x}}^{i},\sigma^{i})=S_{u}(\Omega_{m}({\vec{x}}_{m},\sigma^{i-1}),p_{i})\\ \text{end} \end{array}$$

With $\Omega \left[\vec{x}\left(t\right),\sigma \left(t\right)\right]$ the configuration at an arbitrary point in time, ${\vec{x}}_{m}$ the measured geometry, **σ** the stress, *p* the pressure, *i*_*max*_ the number of pressure increments, *p*_*m*_ the measured pressure and *S*_*u*_ an operator representing an updated Lagrange solver. If the increments of pressure are small in the final iterations, the stress and pressure at the wall will be in equilibrium at the final iteration. Please note that at each iteration of the BIM, the geometry is reset to the initial, measured geometry. In this study, the patient-specific AA diastolic BP was used as measured pressure and applied in 20 increments according to Equation (3).

### 2.3. Fluid Domain

At the inlet of the fluid domain, a generic, time-varying flow profile assuming a heart rate of 75 beats/min was prescribed as boundary condition. This profile was obtained using the 1D wave propagation model described by Bessems et al. (2007). This resulted in a mean inlet flow of 0.96 L/min, which lies within the physiological range found in literature (Cheng et al., 2003). The patient-specific inlet centerline velocity (*v*_*max*_) was calculated from the prescribed inlet flow (*q*_*in*_) and the patient-specific radius of the inlet (*a*_*in*_), assuming a Poiseuille profile, according to Equation (4).


(4) $$\begin{array}{l} v_{max}=\frac{2q_{in}}{\pi a_{in}^{2}} \end{array}$$

A no-slip condition was assigned to the lumen wall and the shear-thinning behavior of blood was modeled using the Carreau model:


(5) $$\begin{array}{l} \eta \left(\overset{∙}{\gamma }\right)=\eta_{\infty }+\left(\eta_{0}-\eta_{\infty }\right){\left(1+{\left(\lambda \overset{∙}{\gamma }\right)}^{2}\right)}^{\left(n-1\right)/2} \end{array}$$

With $\eta \left(\overset{∙}{\gamma }\right)$ the viscosity as a function of shear rate, η_0_ the viscosity at zero shear rate (0.056 Pa·s), η_∞_ the viscosity at infinite shear rate (0.00345 Pa·s), λ the time constant (3.313 s) and *n* the power index (0.3568) (Sequeira and Janela, 2007).

At the outlet of the fluid domain, a 3-element Windkessel (WK) model was used to prescribe the outlet pressure based on the outlet flow. The Windkessel model consists of a characteristic impedance (Z), peripheral resistance (R), and arterial compliance (C). The impedance was calculated by modeling minimal reflections at the outlet, according to Equation (6).


(6) $$\begin{array}{l} Z=\sqrt{\frac{\rho hE}{2\pi^{2}\left(1-\nu^{2}\right)a^{5}}} \end{array}$$

With ρ the fluid density (1,040 kg/m^3^), *h* the wall thickness (2 mm), *E* = 2*G*(1 + ν) the Young's modulus, ν the Poisson's ratio (0.5) and *a* the outlet radius (1 mm) (Bessems et al., 2007). The peripheral resistance was estimated from the total resistance (*R*_*T*_) using Equation (7), with $\bar{p}$ the mean arterial pressure, calculated according to Equation (8), and $\bar{q}$ the mean inlet flow. The compliance was estimated using Equation (9) with τ the RC-time (0.6 s).


(7) $$\begin{array}{l} R_{T}=Z+R=\frac{\bar{p}}{\bar{q}} \end{array}$$

(8) $$\begin{array}{l} \bar{p}=\frac{2}{3}P_{dia}+\frac{1}{3}P_{sys} \end{array}$$

(9) $$\begin{array}{l} C=\frac{\tau }{R} \end{array}$$

Within MATLAB, the WK parameters were optimized by iteratively adjusting the peripheral resistance and compliance to match the patient-specific AA diastolic and systolic BP using the inlet flow profile. To incorporate the effect of the compliance of the AAA geometry on the pressure waveform in this optimization, an additional compliance (*C*_*A*_) was added in front of the 3-element WK model (Figure 1).

In the FSI-PSE workflow, the measured geometry represents the diastolic configuration and the BIM was executed using the AA diastolic BP. After application of the BIM, the AA systolic pressure was applied to obtain the systolic geometry. In the workflow without PSE (FSI-noPSE), the measured geometry was assumed to represent the unloaded and unstressed configuration. The AA diastolic and systolic BP were applied to the measured geometry to obtain the diastolic and systolic geometries. Subsequently, the volumes of the diastolic and systolic geometries were calculated and the arterial compliance was calculated using:


(10) $$\begin{array}{l} C_{A}=\frac{dV}{dP}\approx \frac{V_{sys}-V_{dia}}{P_{sys}-P_{dia}} \end{array}$$

### 2.4. Fluid-Structure Interaction Simulations

The fluid and structural models interact in a partitioned approach using Ansys System Coupling (Ansys Inc., Canonsburg, PA, USA, version 2020R1). Especially in strongly coupled problems, partitioned FSI schemes suffer from a numerical instability known as the added-mass effect. This effect occurs as a result of the solution mismatch between the separate domains, since the fluid forces depend upon the predicted structural displacements instead of the correct ones (Guess et al., 2017). To overcome the added mass instability, the quasi-Newton stabilization algorithm introduced by Degroote et al. (2010) was employed. In this algorithm, the Jacobian is approximated and information from previous time steps is reused, resulting in an accelerated convergence speed compared to other stabilization methods.

Due to the interactions with the structural model, the lumen wall in the fluid model is not rigid, but moves with a certain grid velocity. The arbitrary Lagrange-Euler (ALE) method was used to deform the computational grid such that it follows the movement at the wall, but does not follow the fluid elements in the interior fluid domain (van de Vosse et al., 2003). To maintain the quality of the fluid mesh, the mesh was updated using diffusion-based smoothing and remeshing was enabled (Ansys, 2020).

For each FSI model, a heart rate of 75 beats per minute was assumed. In both the FSI-PSE as FSI-noPSE workflow, the measured geometry was used as starting configuration. The solution was initialized with the AA diastolic BP and three cardiac cycles were modeled to ensure a stable, periodic solution. The last cardiac cycle was used for result evaluation.

### 2.5. Result Analyses

For the solid domain, the total systolic displacement and the systolic Von Mises stress of the AAA wall were evaluated. The measured AAA geometry was used as reference to calculate the systolic displacement. Additionally, the systolic displacement with respect to the diastolic geometry was calculated by subtracting the diastolic displacement, and is referred to as the corrected displacement. For the fluid domain, the flow and pressure were examined. Additionally, τ_*wa*_, the time-averaged WSS (TAWSS) for each point on the AAA wall was calculated using Equation (11), with *T* the cycle period and *t* the total simulation time (Salman et al., 2019). Furthermore, the direction of the WSS over time was quantified using the oscillatory shear index (OSI), according to Equation (12). The OSI ranges between 0 and 0.5. An OSI of 0 indicates that the WSS is unidirectional, whereas an OSI of 0.5 indicates oscillating WSS with a TAWSS equal to zero (Salman et al., 2019). Regions with low TAWSS and high OSI are believed to be prone to ILT formation (Salman et al., 2019).


(11) $$\begin{array}{l} \tau_{wa}=\frac{1}{T}\int_{t-T}^{t}|WSS|\;dt \end{array}$$

(12) $$\begin{array}{l} \text{OSI}=\frac{1}{2}\left(1-\frac{\left|\frac{1}{T}\int_{t-T}^{t}WSS\;dt\right|}{\frac{1}{T}\int_{t-T}^{t}|WSS|\;dt}\right) \end{array}$$

To exclude the effect of differences in systolic pressure between the FSI-PSE and FSI-noPSE simulations in further analyses, the displacement, stress and TAWSS were scaled to the measured patient-specific AA systolic BP, using the systolic BP resulting from the FSI simulation, according to Equation (13), with ϕ the quantity of interest.


(13) $$\begin{array}{l} \phi_{scaled}=\phi \cdot \frac{SBP_{AA}}{SBP_{FSI}} \end{array}$$

Additionally, for each quantity (ϕ), the spatial difference was calculated according to Equation (14), to quantify the difference in quantity value between corresponding nodes in the FSI-PSE and FSI-noPSE simulations.


(14) $$\begin{array}{l} \delta_{s}=\frac{\phi_{scaled,noPSE}-\phi_{scaled,PSE}}{\mu_{\phi_{scaled,PSE}}}\cdot 100\% \end{array}$$

Statistical tests were executed to test the significance of differences in quantities between the FSI-PSE and FSI-noPSE simulations and between the different patient groups. In all cases, two groups were compared using Wilcoxon Signed-rank tests, since the homogeneity of variance and normality assumptions were not met in the presented datasets. Statistical significance was reached when the calculated *p*-value was smaller than 0.05.

## 3. Results

**Figure 3:**
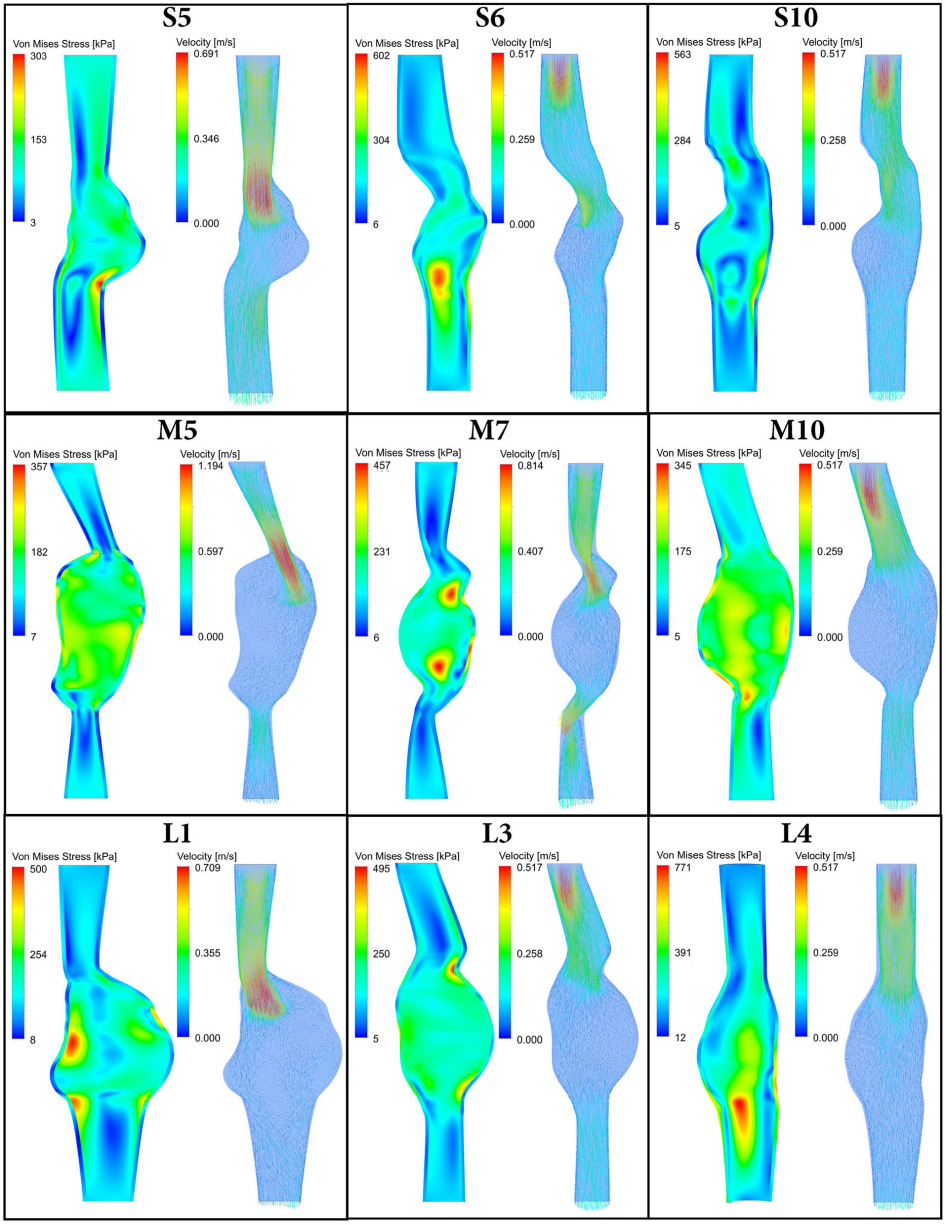
Systolic Von Mises stress **(left)** and systolic velocity vectors **(right)** resulting from FSI simulations with PSE plotted on a longitudinal section of 9 representative patient AAA geometries. Note there is a time delay between systolic velocity (flow) and systolic stress, as illustrated in Figure 4.

For all 30 AAA patients, both FSI-PSE and FSI-noPSE simulations were executed successfully. For nine representative patient datasets, Figure 3 displays the systolic Von Mises stress and systolic velocity vectors on a longitudinal section of the AAA geometry. This figure illustrates the large variety in patient-specific geometries and therefore, the robustness of the obtained FSI framework. These results demonstrate the feasibility of using 3D+t US-based geometries to execute FSI simulations.

The remainder of this section will focus on evaluating the influence of incorporating pre-stress by comparing the FSI-PSE and FSI-noPSE simulations.

### 3.1. Flow and Pressure Waveforms

**Figure 4:**
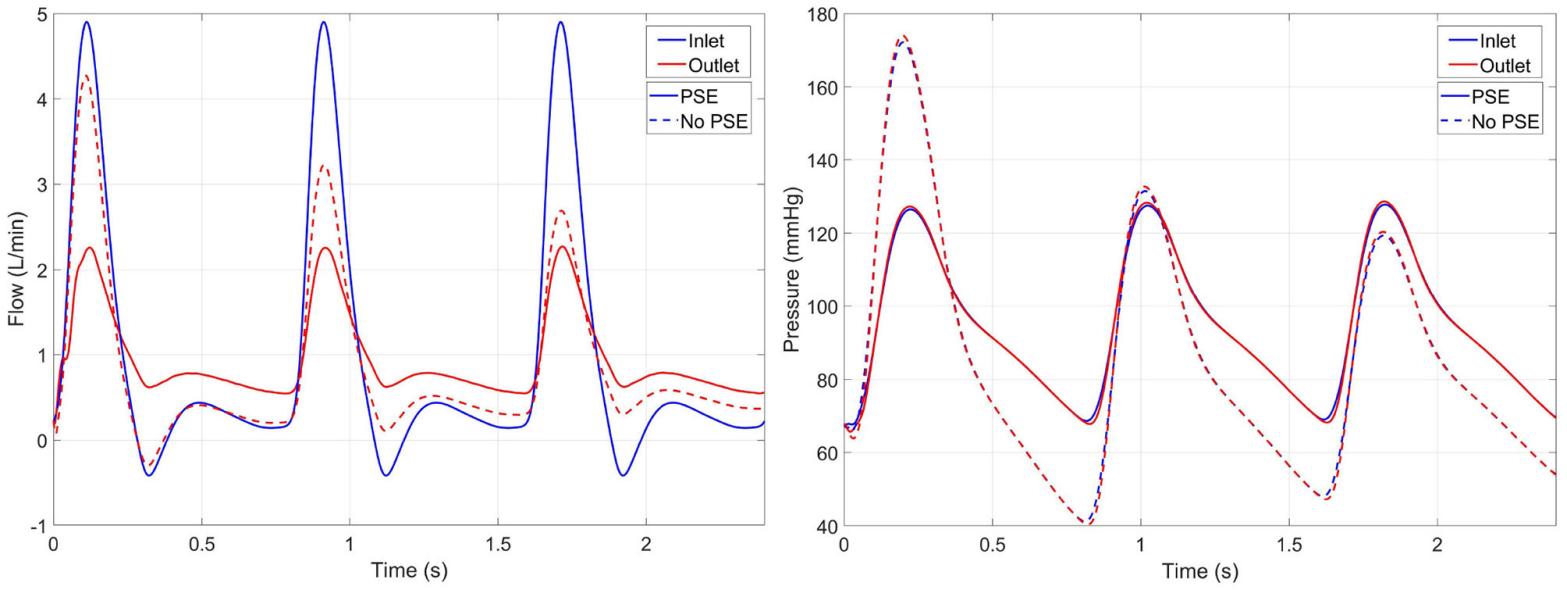
Inlet (blue) and outlet (red) flow **(left)** and pressure **(right)** resulting from the FSI simulations with PSE (solid) and without PSE (dashed) for patient L8. Note that the inlet flow is similar for both simulations, since a generic inlet flow is prescribed.

The flow and pressure waveforms at the inlet and outlet of the domain resulting from the FSI simulations of a representative patient (L8) are visualized in Figure 4. In the FSI-noPSE simulation, the peak flow increased compared to the FSI-PSE simulation, whereas the diastolic flow decreased.

**Figure 5:**
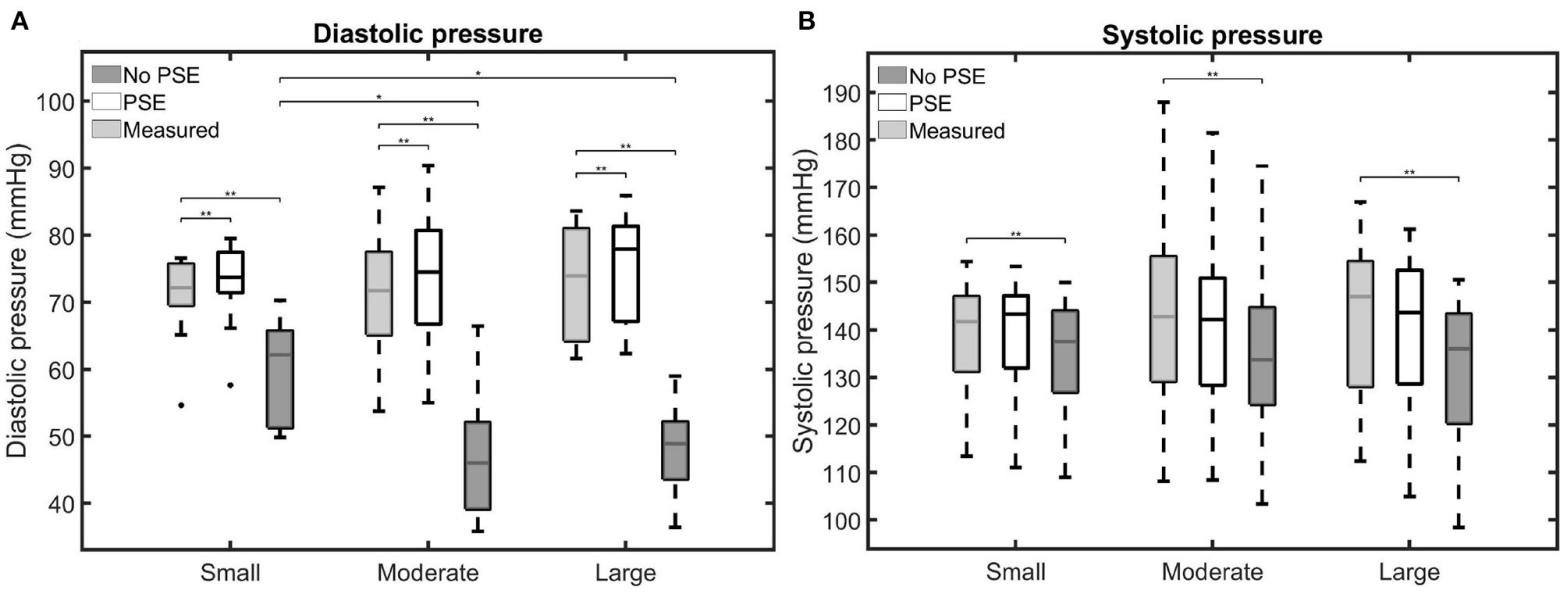
Visualization of the measured and simulated **(A)** diastolic and **(B)** systolic blood pressures for the different patient groups. Significant differences between groups are indicated with bars and stars (^**^p ≤ 1e-2, ^*^p ≤ 0.05).

The measured and simulated diastolic and systolic BP are visualized in Figure 5. Furthermore, for each AAA dataset, the difference between measured and simulated AA diastolic and systolic BP are listed in Supplementary Table 1. Figure 5A shows that the simulated diastolic pressures for all patient groups are significantly different from the measured diastolic pressure. In the FSI simulations with PSE, the diastolic BP is overestimated by 0.4–7.4%, with a mean overestimation of 3.3%. The diastolic BP is underestimated by 7.1–41.7% in the FSI simulation without PSE, with a mean underestimation of 27.6%. For the FSI-PSE simulations, no significant differences in diastolic BP are observed between the patient groups, similar as for the measured diastolic BP. However, for the FSI-noPSE simulations, the diastolic BP for the moderate and large groups were significantly different from the small group.

No significant differences were observed between the measured and FSI-PSE simulated systolic BP (Figure 5B). However, the FSI-noPSE simulated systolic BP did differ significantly from the measured systolic BP. The deviation in systolic BP ranges from 0.1 to 6.6% (mean = 1.7%) and 0.3 to 12.4% (mean = 5.7%) for the FSI-PSE and FSI-noPSE simulations, respectively. The systolic BP is underestimated in all FSI simulations without PSE, whereas it is underestimated in 13 out of 30 (43%) of the FSI simulations with PSE. For both the measured as simulated systolic BP, no significant differences between patient groups were observed.

### 3.2. Structural Domain

**Figure 6:**
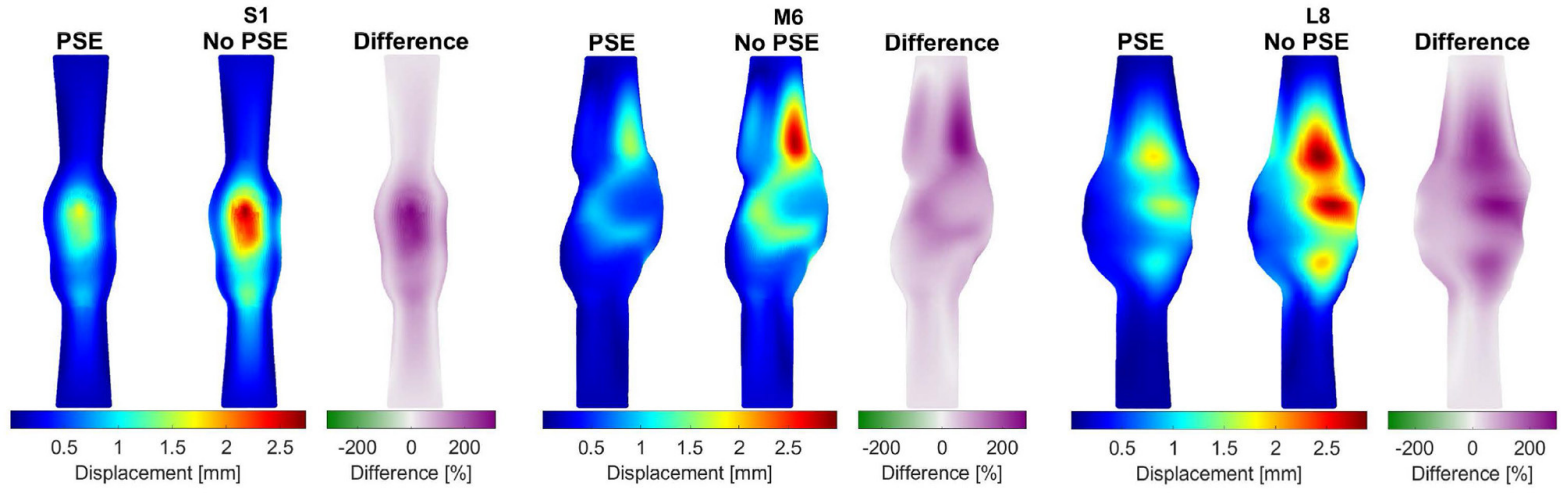
Visualization of the systolic displacement resulting from the FSI simulations with and without PSE, and the difference in displacement for patients S1, M6, and L8.

**Figure 7:**
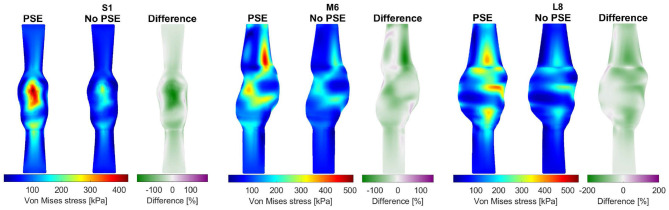
Visualization of the systolic stress resulting from the FSI simulations with and without PSE, and the difference in stress for patients S1, M6, and L8.

For three representative patients, the systolic displacements and stresses resulting from the FSI-PSE and FSI-noPSE simulations, and the corresponding spatial differences, are visualized in Figures 6, 7, respectively. These figures illustrate that regions of high displacement correspond with regions of high wall stress. The pattern of the difference plot closely resembles the pattern of the displacement and stress plots of the separate FSI simulations, i.e., greater differences were observed in regions of high displacement and stress. When the PSE was omitted, the displacement increased in the entire geometry, whereas the stress decreased in the major part of the geometry. At a few locations, the stress was slightly increased compared to the simulation with PSE.

**Figure 8:**
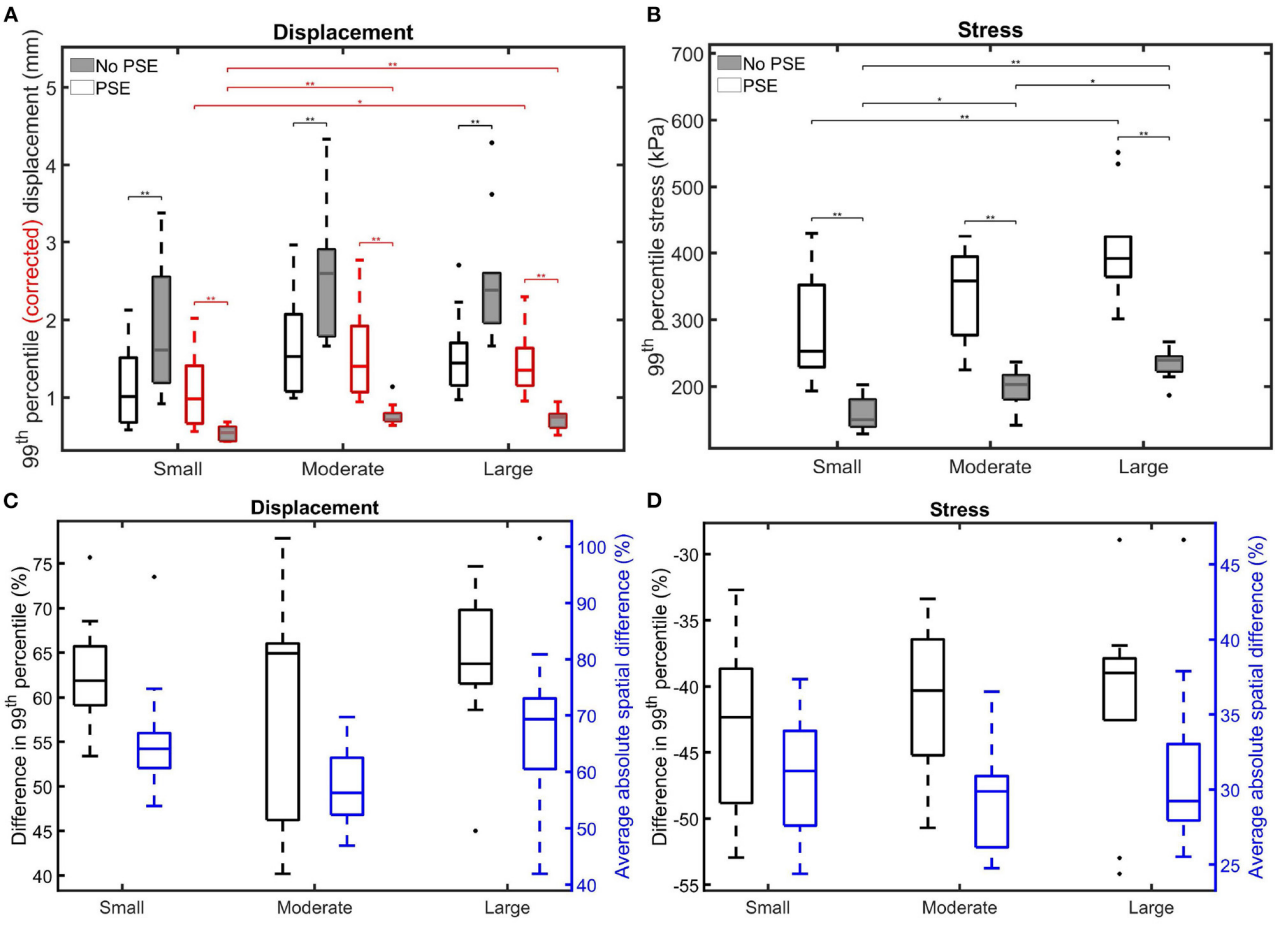
Visualization of the 99th percentile systolic **(A)** (corrected) displacement and **(B)** wall stress for both FSI simulations for the different patient groups. The corrected displacement is shown in red. **(C,D)** Visualize the difference in 99th percentile (black) and average absolute spatial difference (blue) between the FSI simulations for the displacement and stress, respectively. Significant differences between groups are indicated with bars and stars (^**^p ≤ 1e-2, ^*^p ≤ 0.05).

Figure 8 summarizes the findings for the complete patient population and allows for a comparison between the different patient groups. Figure 8A shows the 99th percentile displacement and corrected displacement for both FSI simulations and confirms the increase in displacement when the PSE was omitted, as also seen in Figure 6. However, the corrected displacement decreases in the FSI-noPSE simulation. Furthermore, the range in corrected displacement was reduced compared to the FSI-PSE simulations. For all patient groups, the differences in (corrected) displacement between the FSI-PSE and FSI-noPSE were found to be statistically significant. No significant differences in displacement were observed between the different patient groups. However, for the corrected displacement, the FSI-PSE simulations result in significantly higher corrected displacement for the large groups compared to the small group. For the FSI-noPSE simulations, the corrected displacements of the moderate and large groups were significantly higher compared to the small group. Figure 8C shows that the overall average difference in 99th percentile displacement equals 62.1%. Furthermore, this figure shows that the overall average absolute spatial difference in displacement equals 64.4%, which is in line with the average difference in 99th percentile. For both the difference in 99th percentile as the spatial difference, no significant differences between groups were observed.

Figure 8B clearly shows the decrease in 99th percentile wall stress when the PSE was omitted. Furthermore, the range in stress value decreased compared to the simulation with PSE. For all patient groups, the differences in stress between the FSI-PSE and FSI-noPSE simulations were statistically significant. For both FSI simulations, an increase in stress was observed for increasing maximum diameter. For the FSI-noPSE simulations, the differences between all groups were statistically significant, whereas only the difference between the small and large groups was significant for the FSI-PSE simulations. The overall average decrease in 99^th^ percentile stress (Figure 8D) equals 41.5%, whereas the overall average absolute spatial difference equals 30.7%. For both the difference in 99th percentile as the spatial difference, no significant differences between groups were observed.

### 3.3. Fluid Domain

**Figure 9:**
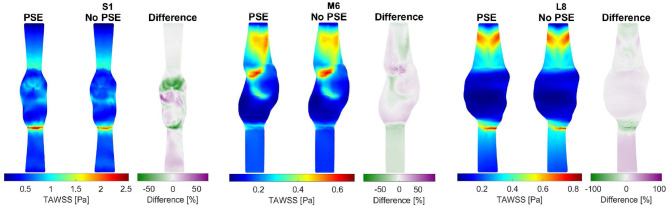
Visualization of the time-averaged wall shear stress (TAWSS) resulting from the FSI simulations with and without PSE, and the difference in TAWSS for patients S1, M6, and L8.

**Figure 10:**
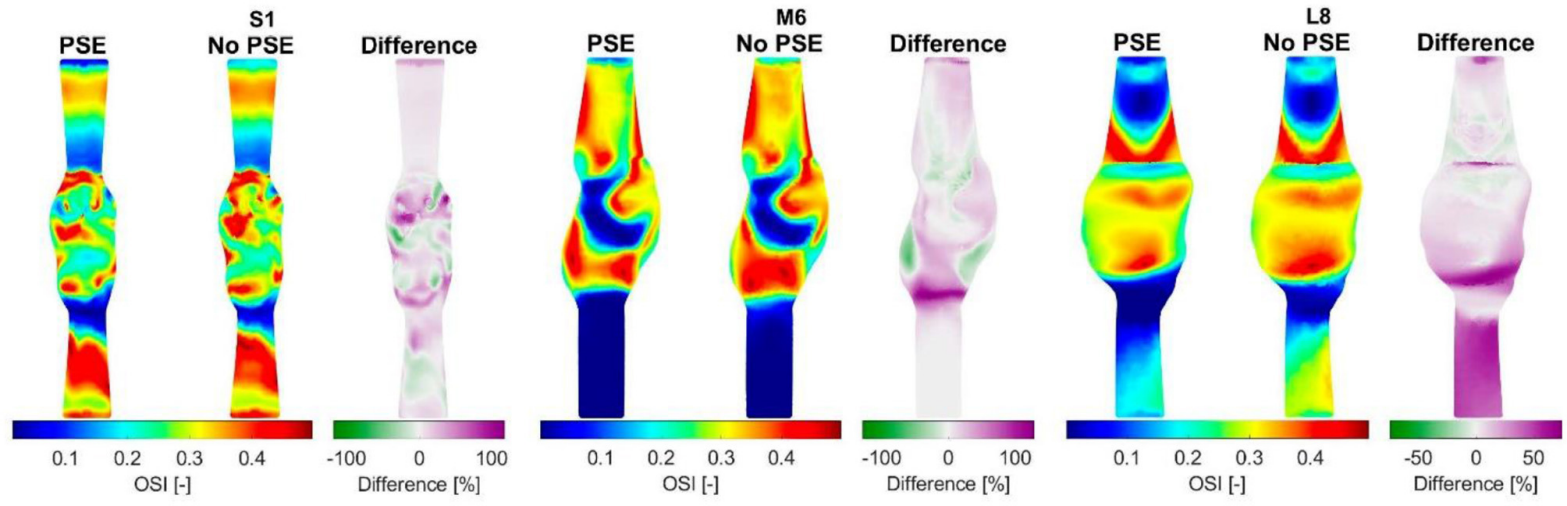
Visualization of the oscillatory shear index (OSI) resulting from the FSI simulations with and without PSE, and the difference in OSI for patients S1, M6, and L8.

For three representative patients, the TAWSS and OSI resulting from the FSI-PSE and FSI-noPSE simulations, and the corresponding spatial differences, are visualized in Figures 9, 10, respectively. These figures show that high TAWSS values mainly occur in the neck of the AAA and at the proximal and distal ends of the aneurysm region. High OSI values are mainly observed in the aneurysm regions, whereas the TAWSS is relatively low in this region. For patient S1, a clear difference in TAWSS and OSI patterns can be seen when the PSE is omitted, which is also reflected in the difference plots. For patient M6, the TAWSS patterns appear highly similar, whereas the OSI patterns do differ slightly. For the TAWSS, the difference plot does show differences, especially in the low TAWSS regions. For patient L8, both TAWSS and OSI patterns appear highly similar. However, the difference plots show some noticeable differences.

**Figure 11:**
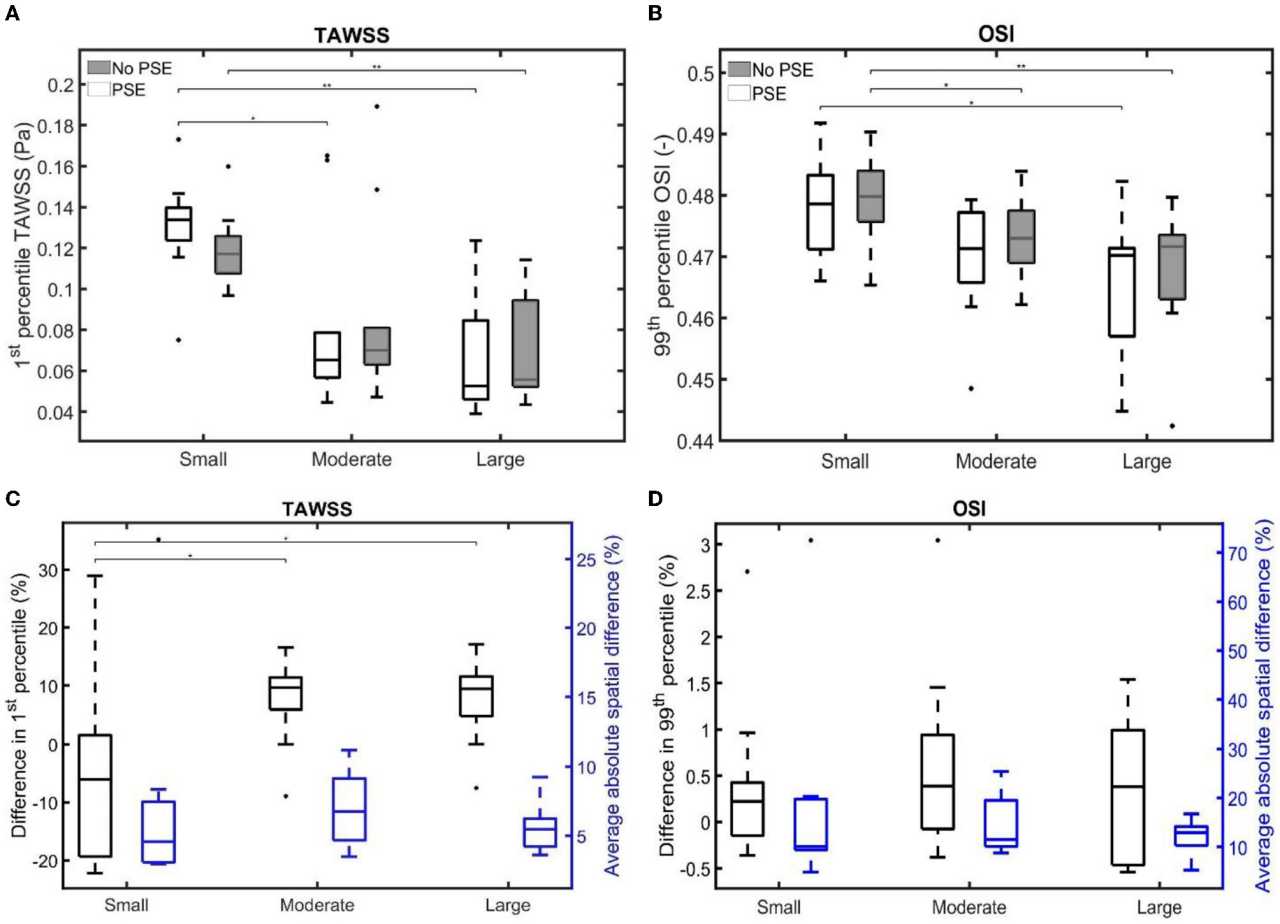
Visualization of **(A)** the 1st percentile TAWSS and **(B)** the 99th percentile OSI for both FSI simulations for the different patient groups. **(C,D)** Visualize the difference in 1st/99th percentile (black) and average absolute spatial difference (blue) between the FSI simulations for the TAWSS and OSI, respectively. Significant differences between groups are indicated with bars and stars (^**^p ≤ 1e-2, ^*^p ≤ 0.05).

For both TAWSS and OSI values, no clear decrease or increase was observed when the PSE was omitted, which is confirmed by Figures 11A,B, respectively. For the FSI-PSE simulations, the TAWSS for the moderate and large groups was significantly decreased compared to the small group. Furthermore, the difference in OSI between the small and large groups was significant. For the FSI-noPSE simulations, only the difference in TAWSS between the small and large group was significant. The OSI for the moderate and large groups was significantly decreased compared to the small group. Figure 11C shows that the overall average difference in 1st percentile TAWSS equals 3.0%, whereas the overall average absolute spatial difference equals 6.5%. The difference in 1st percentile TAWSS value for the moderate and large groups were significantly increased compared to the small group. The overall average difference in 99th percentile OSI values is as small as 0.5%, whereas the overall absolute spatial difference equals 14.7%, as shown in Figure 11D. No significant differences in average absolute spatial differences between groups were found for the OSI.

The 99th/1st percentile values and spatial differences in displacement, stress, TAWSS, and OSI for each individual patient are summarized in Supplementary Tables 2, 3, respectively.

## 4. Discussion

In this study, a robust, automated framework to execute FSI simulations of AAAs based on 3D+t US data was developed. The framework was successfully employed on an extensive dataset of 30 AAA patients with maximum diameters ranging from 27 to 56 mm. Furthermore, the effect of incorporating the PSE on the wall mechanics and hemodynamics was investigated.

Besides a high variety in maximum AAA diameter, the presented data set also reflects a high variety in BP, with diastolic BP ranging from 54.6 to 84.5 mmHg and systolic BP ranging from 108.2 to 188.0 mmHg. As shown in Figures 4, 5 and Supplementary Table 1, the FSI simulations with PSE resulted in diastolic and systolic pressures that correspond well with the measured AA pressure, with an average overestimation of the diastolic BP with 3.3% and an average difference of 1.7% in systolic BP. Although the difference in diastolic BP was small, statistical significance was reached. For the systolic BP, no significant difference was observed compared to the measured systolic BP. When the PSE was omitted, the average underestimation of the diastolic and systolic BP equalled 27.6 and 5.7%, respectively. Omitting the PSE resulted in significantly different diastolic and systolic BP compared to the measured BP. In the FSI simulation without PSE, the measured geometry was assumed to be unloaded. In the first step of the FSI simulation, the geometry was initialized with the diastolic BP, which resulted in a large displacement of the AAA wall, causing a decrease in pressure, which resulted in a system that is out of equilibrium. Due to the initial deviation in displacement and pressure, the pressures in the equilibrium state deviate from the desired pressures. Furthermore, it is uncertain if the FSI-noPSE simulations reached their equilibrium state after three cardiac cycles, whereas the FSI-PSE simulations reached a stable, periodic solution after one cardiac cycle. Due to limitations in simulation time, only three cardiac cycles were simulated in this study. When employing the FSI-noPSE framework in future studies, additional cardiac cycles need to be simulated to ensure a stable, periodic solution.

As illustrated in Figure 4, the peak flow increased in the simulation without PSE and the diastolic flow decreased, which indicates a less compliant AAA wall when the PSE was omitted. Although the systolic displacement significantly increases when the PSE was omitted (Figures 6, 8A,C), the corrected displacement significantly decreased, which explains the impaired compliance that was observed in Figure 4.

When PSE was omitted, the 99th percentile systolic displacement increased with an average of 62.1%, whereas the 99th percentile systolic stress decreased with an average of 41.5%. The average absolute spatial difference equals 64.4 and 30.7% for the displacement and stress, respectively. These spatial differences closely resemble the differences in 99th percentile values, indicating that the displacement and stress patterns in the different FSI simulations closely resemble each other, as also observed in Figures 6, 7. The increase in displacement is caused by the increase in additional load. In the FSI-PSE simulations, the stresses at diastolic pressure were already incorporated in the geometry, so the additional load that was applied equals the difference between the systolic and diastolic pressure. In the FSI-noPSE simulations, no pre-stress was incorporated in the geometry, so the additional load equals the systolic pressure. Due to the larger displacements in the FSI-noPSE simulations, the geometry tends to become smoother compared to the FSI-PSE simulations (de Putter et al., 2007). Due to the loss of curvature in the geometry, the stress decreases when PSE was omitted. Furthermore, the range in corrected displacement (Figure 8A) and wall stress (Figure 8B) decreases due to the loss of curvature. In both FSI simulations, the 99th percentile wall stress increased with increasing maximum diameter, which is in agreement with literature (van Disseldorp et al., 2018).

For both the TAWSS and OSI, no clear decrease or increase was observed when the PSE was omitted (Figures 9–11). However, differences in TAWSS and OSI patterns were observed and quantified by calculating the spatial differences. The average absolute spatial difference in TAWSS ranges from 3.0 to 26.4% with an average of 6.5%. The average absolute spatial difference in OSI ranges from 4.8 to 72.6% with an average of 14.7%. Although the differences in 1st percentile TAWSS and 99th percentile OSI are small, the increased spatial difference indicates that the patterns are different, especially for the OSI. Therefore, omitting the PSE may cause the regions that are prone to ILT formation (low TAWSS, high OSI) to deviate from the regions detected in the simulation with PSE.

The main limitations of this study result from simplifications made to the current FSI model. Firstly, a generic inlet flow with a generic heart rate, combined with a Poiseuille profile, was used as boundary condition. In future models, the patient-specific flow profile should be obtained, for example with US Doppler or Vector Velocity Imaging, and used as boundary condition to yield a more patient-specific approach. Secondly, the aorto-iliac bifurcation was omitted, since the iliac arteries are difficult to visualize using 3D+t US. In the past, it has been shown that the presence of the aortic bifurcation does not significantly influence the wall mechanics in the AAA region (van Disseldorp et al., 2016). However, previous studies have shown that the bifurcation geometry does influence the hemodynamics in the AAA region (Li and Kleinstreuer, 2007; Drewe et al., 2017). In a future study, the influence of including the bifurcation geometry on the wall mechanics and hemodynamics should be investigated. Thirdly, a generic shear modulus, based on maximum AAA diameter, was used for the AAA wall. For a more patient-specific approach, the patient-specific shear modulus could be obtained by using 3D speckle tracking to determine the displacements of the AAA wall over the cardiac cycle and matching the displacements found in the CSS model to these displacements, as proposed by van Disseldorp et al. (2018). Based on this future incentive, the simple, Neo-Hookean material model was used in this study. The usage of more complex material models is believed to lead to higher parameter uncertainty when estimating patient-specific material properties. Furthermore, the difference in displacement between the FSI-PSE and FSI-noPSE simulations is expected to further increase when a non-linear material model is used for the FSI-noPSE simulation, since the vessel wall is more compliant in the lower pressure regime of the non-linear model (Holzapfel et al., 2012). Lastly, the wall thickness was assumed to be homogeneous, since the local wall thickness cannot be assessed by conventional imaging. However, previous studies have shown that the wall thickness significantly influences the wall stress values and distribution (Scotti et al., 2005; Scotti and Finol, 2007; Xenos and Bluestein, 2011; van Disseldorp et al., 2018). Improvements in US imaging are required before the patient-specific outer wall geometry can be included in the model.

To conclude, this study is the first to successfully execute 3D+t US-based FSI simulations with PSE on an extensive set of patient data and to quantify the influence of the PSE on wall mechanics and hemodynamics. FSI simulations with PSE resulted in simulated pressures that deviated 3.3 and 1.7% from the measured diastolic and systolic BP, respectively, compared to deviations of 27.6% (diastolic) and 5.7% (systolic) for the FSI simulations without PSE. Furthermore, omitting the prestress yields increased systolic displacements (40.2–77.8%) and decreased systolic wall stresses (28.9–54.2%). No clear increase or decrease in TAWSS or OSI was observed. However, average spatial differences of 6.5 and 14.7% were found for the TAWSS and OSI, respectively, indicating that the TAWSS and OSI patterns are dissimilar. These results underline the importance of incorporating pre-stress in FSI simulations, especially for the wall mechanics. After validation, the obtained framework to execute 3D+t US-based FSI simulations provides an important tool for personalized modeling of AAAs as well as longitudinal studies on AAA growth, ILT formation and rupture risk.

## Data Availability Statement

The raw data supporting the conclusions of this article will be made available by the authors, without undue reservation.

## Ethics Statement

The studies involving human participants were reviewed and approved by Medical research Ethics Committees United (MEC-U). The patients/participants provided their written informed consent to participate in this study.

## Author Contributions

JF developed the FSI framework and executed the simulations and analyses. EM and AN developed the automatic segmentation toolbox and provided the 3D+t US segmentations. EM, AN, MS, FV, and RL were involved to critically review the progress and manuscript. All authors contributed to the article and approved the submitted version.

## Conflict of Interest

The authors declare that the research was conducted in the absence of any commercial or financial relationships that could be construed as a potential conflict of interest.

## Publisher's Note

All claims expressed in this article are solely those of the authors and do not necessarily represent those of their affiliated organizations, or those of the publisher, the editors and the reviewers. Any product that may be evaluated in this article, or claim that may be made by its manufacturer, is not guaranteed or endorsed by the publisher.
