# Corrigendum: Ultrasound-Based Fluid-Structure Interaction Modeling of Abdominal Aortic Aneurysms Incorporating Pre-Stress

**DOI:** 10.3389/fphys.2022.885959

**Published:** 2022-04-19

**Authors:** Judith H. C. Fonken, Esther J. Maas, Arjet H. M. Nievergeld, Marc R. H. M. van Sambeek, Frans N. van de Vosse, Richard G. P. Lopata

**Affiliations:** ^1^ Photoacoustics and Ultrasound Laboratory Eindhoven (PULS/e), Department of Biomechanical Engineering, Eindhoven University of Technology, Eindhoven, Netherlands; ^2^ Department of Vascular Surgery, Catharina Hospital Eindhoven, Eindhoven, Netherlands; ^3^ Cardiovascular Biomechanics, Department of Biomechanical Engineering, Eindhoven University of Technology, Eindhoven, Netherlands

**Keywords:** abdominal aortic aneurysms, patient-specific, fluid-structure interaction modeling, rupture risk, prestress estimation, wall mechanics, hemodynamics

In the original article, there was an error in the calculation of the Oscillatory Shear Index (OSI). The corrected Figure 10 appears below.

**FIGURE 10:**
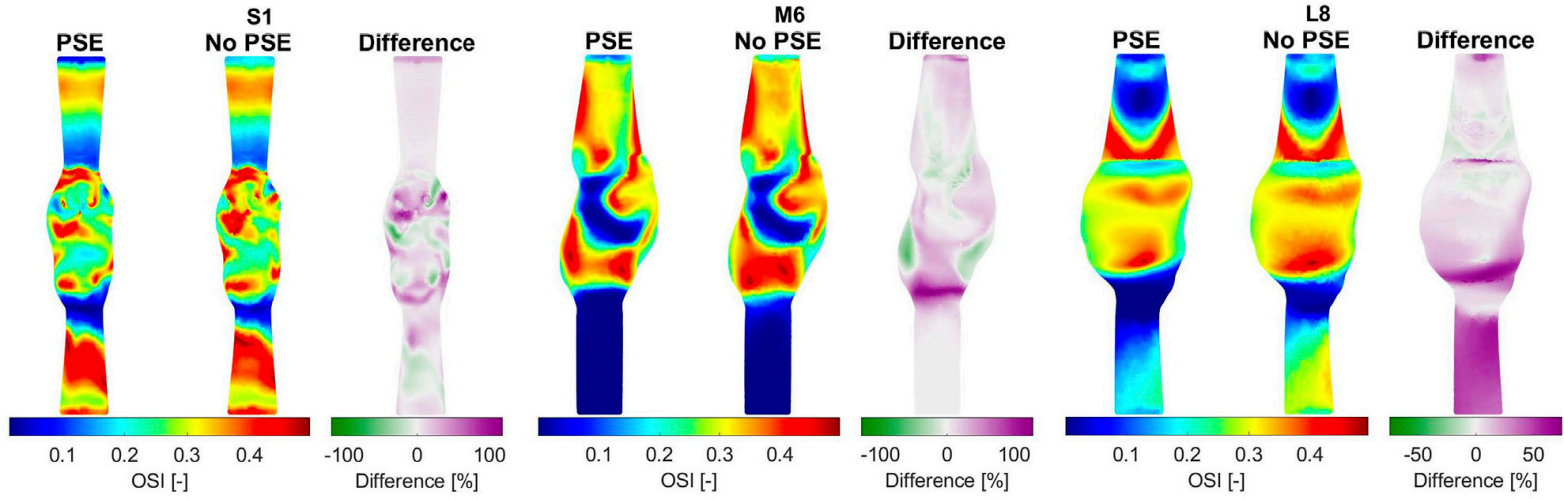
Visualization of the oscillatory shear index (OSI) resulting from the FSI simulations with and without PSE, and the difference in OSI for patients S1, M6, and L8.

The corrected Figure 11 appears below.

**FIGURE 11:**
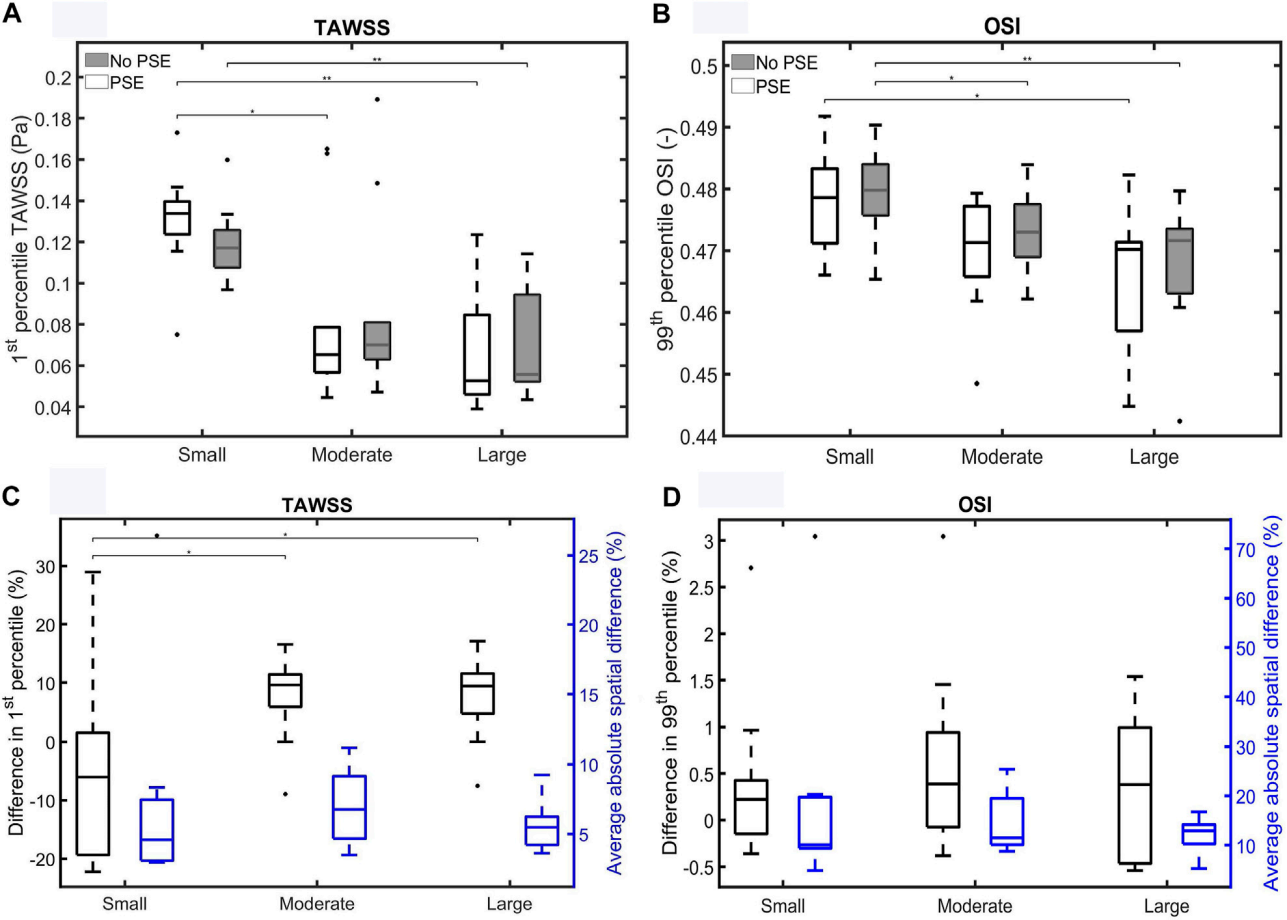
Visualization of **(A)** the 1st percentile TAWSS and **(B)** the 99th percentile OSI for both FSI simulations for the different patient groups. **(C,D)** Visualize the difference in 1st/99th percentile (black) and average absolute spatial difference (blue) between the FSI simulations for the TAWSS and OSI, respectively. Significant differences between groups are indicated with bars and stars (**p ≤ 1e-2, *p ≤ 0.05).

A correction has been made to **3. Results**, “3.3 Fluid Domain”. The corrected section appears below:

“For three representative patients, the TAWSS and OSI resulting from the FSI-PSE and FSI-noPSE simulations, and the corresponding spatial differences, are visualized in **Figures 9, 10**, respectively. These figures show that high TAWSS values mainly occur in the neck of the AAA and at the proximal and distal ends of the aneurysm region. High OSI values are mainly observed in the aneurysm regions, whereas the TAWSS is relatively low in this region. For patient S1, a clear difference in TAWSS and OSI patterns can be seen when the PSE is omitted, which is also reflected in the difference plots. For patient M6, the TAWSS patterns appear highly similar, whereas the OSI patterns do differ slightly. For the TAWSS, the difference plot does show differences, especially in the low TAWSS regions. For patient L8, both TAWSS and OSI patterns appear highly similar. However, the difference plots show some noticeable differences.

For both TAWSS and OSI values, no clear decrease or increase was observed when the PSE was omitted, which is confirmed by **Figures 11A,B**, respectively. For the FSI-PSE simulations, the TAWSS for the moderate and large groups was significantly decreased compared to the small group. Furthermore, the difference in OSI between the small and large groups was significant. For the FSI-noPSE simulations, only the difference in TAWSS between the small and large group was significant. The OSI for the moderate and large groups was significantly decreased compared to the small group. **Figure 11C** shows that the overall average difference in 1st percentile TAWSS equals 3.0%, whereas the overall average absolute spatial difference equals 6.5%. The difference in 1st percentile TAWSS value for the moderate and large groups were significantly increased compared to the small group. The overall average difference in 99th percentile OSI values is as small as 0.5%, whereas the overall absolute spatial difference equals 14.7%, as shown in **Figure 11D**. No significant differences in average absolute spatial differences between groups were found for the OSI.

The 99th/1st percentile values and spatial differences in displacement, stress, TAWSS, and OSI for each individual patient are summarized in **Supplementary Tables S2, S3**, respectively.”

A correction has been made to **4. Discussion**, paragraph 5. The corrected paragraph appears below:

“For both the TAWSS and OSI, no clear decrease or increase was observed when the PSE was omitted (**Figures 9–11**). However, differences in TAWSS and OSI patterns were observed and quantified by calculating the spatial differences. The average absolute spatial difference in TAWSS ranges from 3.0 to 26.4% with an average of 6.5%. The average absolute spatial difference in OSI ranges from 4.8 to 72.6% with an average of 14.7%. Although the differences in 1st percentile TAWSS and 99th percentile OSI are small, the increased spatial difference indicates that the patterns are different, especially for the OSI. Therefore, omitting the PSE may cause the regions that are prone to ILT formation (low TAWSS, high OSI) to deviate from the regions detected in the simulation with PSE.”

A correction has been made to **4. Discussion**, paragraph 7. The corrected paragraph appears below:

“To conclude, this study is the first to successfully 3D+t US-based FSI simulations with PSE on an extensive set of patient data and to quantify the influence of the PSE on wall mechanics and hemodynamics. FSI simulations with PSE resulted in simulated pressures that deviated 3.3 and 1.7% from the measured diastolic and systolic BP, respectively, compared to deviations of 27.6% (diastolic) and 5.7% (systolic) for the FSI simulations without PSE. Furthermore, omitting the prestress yields increased systolic displacements (40.2–77.8%) and decreased systolic wall stresses (28.9–54.2%). No clear increase or decrease in TAWSS or OSI was observed. However, average spatial differences of 6.5 and 14.7% were found for the TAWSS and OSI, respectively, indicating that the TAWSS and OSI patterns are dissimilar. These results underline the importance of incorporating pre-stress in FSI simulations, especially for the wall mechanics. After validation, the obtained framework to execute 3D+t US-based FSI simulations provides an important tool for personalized modeling of AAAs as well as longitudinal studies on AAA growth, ILT formation and rupture risk.”

The authors apologize for these errors and state that this does not change the scientific conclusions of the article in any way. The original article has been updated.

