# Supplementary material for: Ultrasound-Based Fluid-Structure Interaction Modeling of Abdominal Aortic Aneurysms Incorporating Pre-stress
Source: Front Physiol. 2021 Aug 13;12:717593. doi: 10.3389/fphys.2021.717593 (PMC8414835; doi:10.3389/fphys.2021.717593)
Supplement: Supplementary file 1 [file Data_Sheet_1.PDF]

frontiers

Supplementary Material

1 SUPPLEMENTARY TABLES AND FIGURES

1.1 Tables

Table S1. Summary of the abdominal aortic (AA) diastolic blood pressure (P_{dia}) and systolic blood pressure (P_{sys}) and the difference between measured (AA) BP and simulated BP for both the FSI-PSE and FSI-noPSE simulations for all 30 patients and the (group) average (μ). The differences are color coded: green: 0-5%, yellow: 5-10%, red: >15%.

	AA BP (mmHg)		Differences FSI-PSE (%)		Differences FSI-noPSE (%)			AA BP (mmHg)		Differences FSI-PSE (%)		Differences FSI-noPSE (%)			AA BP (mmHg)		Differences FSI-PSE (%)		Differences FSI-noPSE (%)	
	P_{dia}	P_{sys}	P_{dia}	P_{sys}	P_{dia}	P_{sys}		P_{dia}	P_{sys}	P_{dia}	P_{sys}	P_{dia}	P_{sys}		P_{dia}	P_{sys}	P_{dia}	P_{sys}	P_{dia}	P_{sys}
**S1**	70.4	148.1	3.9	0.5	-10.6	-2.5	**M1**	70.4	155.4	6.7	-2.9	-26.1	-6.9	**L1**	64.2	144.9	4.5	-1.5	-32.8	-4.6
**S2**	75.7	147.0	2.8	0.1	-7.1	-2.1	**M2**	87.1	188.0	3.7	-3.4	-40.4	-7.2	**L2**	61.6	115.5	1.2	1.3	-21.8	-4.0
**S3**	73.0	128.1	3.9	-1.4	-16.0	-5.9	**M3**	53.7	108.2	2.4	0.2	-33.0	-4.4	**L3**	80.1	143.9	0.8	0.6	-34.9	-7.1
**S4**	54.6	113.4	5.5	-2.1	-7.4	-3.9	**M4**	68.6	142.8	2.2	0.2	-37.3	-4.3	**L4**	83.6	154.4	1.7	1.0	-29.4	-7.1
**S5**	72.2	135.5	2.6	0.4	-8.9	-2.5	**M5**	77.4	142.8	4.2	-1.1	-28.4	-7.1	**L5**	76.6	154.4	3.9	-1.2	-33.8	-6.6
**S6**	75.7	146.0	2.3	0.5	-29.1	-5.6	**M6**	75.7	152.3	5.2	-5.0	-39.6	-11.6	**L6**	82.7	167.0	3.8	-3.4	-40.1	-9.8
**S7**	65.1	131.3	1.6	0.5	-23.5	-3.3	**M7**	84.5	156.5	1.9	1.0	-21.4	-4.1	**L7**	81.0	149.1	0.4	1.5	-31.2	-5.8
**S8**	76.6	154.4	3.8	-0.7	-12.7	-2.8	**M8**	59.0	128.1	7.4	-5.9	-39.3	-8.7	**L8**	67.8	128.1	2.1	0.4	-28.9	-6.1
**S9**	72.2	139.7	1.7	2.0	-11.0	-0.3	**M9**	65.1	129.2	2.5	-0.6	-40.0	-3.8	**L9**	71.3	150.2	7.0	-5.2	-38.9	-10.9
**S10**	69.5	143.9	2.7	0.3	-26.3	-4.5	**M10**	73.0	138.6	1.1	0.2	-36.6	-5.5	**L10**	62.5	112.4	6.7	-6.6	-41.7	-12.4
**μS**	**70.5**	**138.7**	**3.1**	**0.0**	**-15.3**	**-3.4**	**μM**	**71.5**	**144.2**	**3.7**	**-1.7**	**-34.2**	**-6.4**	**μL**	**73.1**	**142.0**	**3.2**	**-1.3**	**-33.4**	**-7.4**
**μ**	**71.7**	**141.6**	**3.3**	**-1.0**	**-27.6**	**-5.7**														

Table S2. 99th percentile displacement, corrected displacement, stress and OSI and 1st percentile TAWSS values for FSI simulations with and without PSE

	Displacement (mm)		Corrected displacement (mm)		Stress (kPa)		TAWSS (Pa)		OSI (-)	
	PSE	No-PSE	PSE	No-PSE	PSE	No-PSE	PSE	No-PSE	PSE	No-PSE
**S1**	1.36	2.19 (61.0%)	1.29	0.69 (-47.0%)	321	174 (-45.7%)	0.13	0.11 (-19.3%)	0.49	0.49 (0.1%)
**S2**	0.64	1.06 (64.9%)	0.62	0.44 (-30.2%)	193	129 (-33.4%)	0.15	0.12 (-20.5%)	0.49	0.49 (-0.3%)
**S3**	1.51	2.55 (68.5%)	1.41	0.64 (-54.7%)	352	180 (-48.8%)	0.13	0.13 (-0.1%)	0.48	0.48 (0.0%)
**S4**	0.92	1.53 (65.7%)	0.87	0.56 (-35.6%)	237	137 (-42.3%)	0.13	0.11 (-18.1%)	0.49	0.49 (-0.2%)
**S5**	0.68	1.19 (75.7%)	0.67	0.45 (-32.6%)	229	140 (-38.8%)	0.14	0.11 (-22.2%)	0.49	0.48 (-1.4%)
**S6**	2.13	3.38 (59.1%)	2.02	0.62 (-69.2%)	430	202 (-52.9%)	0.12	0.12 (1.8%)	0.50	0.49 (-0.8%)
**S7**	1.04	1.70 (62.3%)	1.00	0.44 (-55.7%)	254	146 (-42.4%)	0.12	0.13 (1.6%)	0.48	0.49 (0.2%)
**S8**	0.58	0.92 (57.7%)	0.56	0.44 (-22.3%)	213	143 (-32.7%)	0.14	0.13 (-4.4%)	0.49	0.49 (0.1%)
**S9**	0.98	1.50 (53.4%)	0.97	0.54 (-44.5%)	251	154 (-38.7%)	0.17	0.16 (-7.6%)	0.49	0.49 (0.0%)
**S10**	1.65	2.67 (61.4%)	1.56	0.59 (-62.3%)	380	184 (-51.7%)	0.08	0.10 (29.0%)	0.49	0.49 (0.4%)
**MeanS**	**1.15**	**1.87 (63.0%)**	**1.10**	**0.54 (-45.4%)**	**286**	**159 (-42.7%)**	**0.13**	**0.12 (-6.0%)**	**0.49**	**0.49 (-0.2%)**
**M1**	2.07	2.90 (40.2%)	1.92	1.14 (-40.6%)	394	237 (-40.0%)	0.08	0.08 (-0.0%)	0.49	0.49 (0.4%)
**M2**	1.30	1.97 (51.2%)	1.21	0.64 (-47.2%)	400	219 (-45.2%)	0.07	0.08 (11.2%)	0.49	0.49 (-0.2%)
**M3**	1.01	1.66 (65.0%)	0.99	0.67 (-32.1%)	225	142 (-36.9%)	0.04	0.05 (5.9%)	0.48	0.48 (0.1%)
**M4**	2.16	3.07 (42.2%)	2.09	0.79 (-62.0%)	351	204 (-42.0%)	0.06	0.06 (11.4%)	0.49	0.49 (-0.1%)
**M5**	0.99	1.76 (77.8%)	0.94	0.70 (-26.1%)	267	178 (-33.4%)	0.16	0.15 (-8.9%)	0.49	0.49 (-0.1%)
**M6**	1.48	2.58 (74.2%)	1.34	0.74 (-45.3%)	365	216 (-40.7%)	0.06	0.07 (8.5%)	0.48	0.48 (-0.0%)
**M7**	1.57	2.61 (65.9%)	1.55	0.90 (-41.8%)	317	202 (-36.4%)	0.17	0.19 (14.5%)	0.49	0.49 (-0.6%)
**M8**	1.66	2.73 (64.9%)	1.46	0.72 (-50.6%)	377	197 (-47.7%)	0.07	0.07 (6.0%)	0.49	0.49 (-0.3%)
**M9**	2.96	4.33 (46.2%)	2.77	0.71 (-74.4%)	425	210 (-50.7%)	0.06	0.07 (16.5%)	0.49	0.49 (0.1%)
**M10**	1.08	1.79 (66.0%)	1.07	0.70 (-34.7%)	277	181 (-34.5%)	0.05	0.05 (10.7%)	0.48	0.48 (-0.8%)
**MeanM**	**1.63**	**2.54 (59.4%)**	**1.54**	**0.77 (-45.5%)**	**340**	**199 (-40.8%)**	**0.08**	**0.09 (7.6%)**	**0.49**	**0.49 (-0.1%)**
**L1**	1.70	2.47 (45.0%)	1.63	0.95 (-42.1%)	383	238 (-37.9%)	0.12	0.11 (-7.5%)	0.49	0.49 (0.3%)
**L2**	0.97	1.66 (71.3%)	0.95	0.61 (-35.5%)	301	187 (-38.0%)	0.07	0.08 (9.7%)	0.49	0.49 (-0.3%)
**L3**	1.16	1.96 (69.8%)	1.15	0.79 (-31.8%)	313	223 (-28.9%)	0.05	0.05 (4.8%)	0.49	0.49 (0.1%)
**L4**	2.23	3.62 (62.8%)	2.11	0.52 (-75.6%)	551	259 (-53.0%)	0.08	0.09 (11.5%)	0.48	0.48 (-1.1%)
**L5**	1.49	2.52 (69.5%)	1.41	0.74 (-47.2%)	425	244 (-42.6%)	0.10	0.11 (5.8%)	0.49	0.49 (-0.3%)
**L6**	1.40	2.29 (63.8%)	1.30	0.76 (-41.2%)	423	267 (-36.9%)	0.05	0.06 (12.2%)	0.48	0.49 (0.6%)
**L7**	1.05	1.83 (74.7%)	1.03	0.59 (-43.3%)	368	229 (-37.9%)	0.05	0.05 (9.1%)	0.48	0.49 (0.3%)
**L8**	1.59	2.60 (63.8%)	1.53	0.80 (-47.6%)	364	214 (-41.2%)	0.05	0.05 (17.1%)	0.49	0.49 (-0.4%)
**L9**	1.39	2.25 (61.5%)	1.26	0.73 (-42.3%)	401	241 (-39.9%)	0.05	0.05 (0.0%)	0.49	0.49 (-0.2%)
**L10**	2.70	4.28 (58.6%)	2.30	0.75 (-67.3%)	534	245 (-54.2%)	0.04	0.04 (11.6%)	0.49	0.49 (-0.4%)
**MeanL**	**1.57**	**2.55 (64.1%)**	**1.47**	**0.72 (-47.4%)**	**406**	**235 (-41.0%)**	**0.07**	**0.07 (7.4%)**	**0.49**	**0.49 (-0.1%)**
**Mean**	**1.45**	**2.32 (62.1%)**	**1.37**	**0.68 (-46.1%)**	**344**	**197 (-41.5%)**	**0.09**	**0.09 (3.0%)**	**0.49**	**0.49 (-0.2%)**

Table S3. Mean and standard deviation of the absolute spatial differences in displacement, corrected displacement, stress, TAWSS and OSI

	Spatial difference (%)									
	Displacement		Corrected displacement		Stress		TAWSS		OSI	
	Mean	STD	Mean	STD	Mean	STD	Mean	STD	Mean	STD
**S1**	63.1	54.3	39.1	40.6	32.8	28.1	7.5	9.4	48.0	75.9
**S2**	66.1	43.7	26.1	21.4	26.0	16.0	5.6	6.9	28.9	48.8
**S3**	94.6	65.8	51.4	43.9	37.4	32.8	3.0	2.5	27.3	44.3
**S4**	66.9	48.2	31.3	25.7	29.7	22.5	3.0	3.2	22.4	38.9
**S5**	74.8	51.5	29.7	25.4	27.6	19.8	4.5	6.4	39.2	67.3
**S6**	62.6	54.1	64.4	63.1	34.2	33.1	4.6	4.6	17.9	27.5
**S7**	60.7	49.1	54.4	46.3	28.4	21.3	3.1	2.4	22.0	41.1
**S8**	53.9	37.8	19.4	17.9	24.4	13.7	3.3	4.9	24.3	41.8
**S9**	54.9	39.3	38.5	29.6	33.6	20.1	8.3	9.8	32.4	48.7
**S10**	65.1	53.1	62.4	54.8	33.9	31.8	26.4	17.2	49.2	68.5
**MeanS**	**66.3**	**49.7**	**41.7**	**36.9**	**30.8**	**23.9**	**6.9**	**6.7**	**31.2**	**50.3**
**M1**	49.1	35.9	39.7	33.7	30.9	23.4	11.2	9.9	40.5	59.1
**M2**	52.4	33.8	61.6	42.2	30.0	22.7	5.4	4.5	20.8	38.0
**M3**	54.6	56.4	28.9	31.2	25.7	16.5	3.5	6.4	10.5	19.2
**M4**	46.9	39.2	59.7	52.4	30.6	22.5	4.3	4.2	22.0	37.5
**M5**	69.7	60.1	18.7	20.4	24.8	16.1	9.1	9.3	33.7	55.6
**M6**	69.2	52.7	50.4	38.4	29.8	22.4	6.5	6.0	31.4	51.7
**M7**	57.0	59.5	37.5	43.6	26.6	19.9	9.1	10.8	36.0	47.4
**M8**	62.1	48.7	61.4	52.0	33.3	26.7	7.0	7.7	21.9	41.8
**M9**	55.6	48.2	76.0	84.0	36.5	30.7	9.8	14.5	27.4	41.0
**M10**	62.5	47.1	34.9	29.9	26.2	16.0	4.7	8.9	16.6	30.2
**MeanM**	**57.9**	**48.2**	**46.9**	**42.8**	**29.4**	**21.7**	**7.1**	**8.2**	**26.1**	**42.1**
**L1**	41.9	37.2	41.3	39.1	27.9	20.5	5.5	7.9	25.0	43.1
**L2**	73.0	55.0	41.7	33.8	28.6	20.0	4.0	3.5	13.4	25.0
**L3**	67.5	50.1	29.8	23.4	25.5	14.6	4.2	5.8	10.5	22.0
**L4**	80.8	70.1	70.6	75.9	37.9	35.1	3.6	3.3	28.7	50.4
**L5**	62.4	53.9	45.0	42.0	31.5	23.9	5.5	6.4	15.8	28.8
**L6**	60.5	45.2	41.9	40.6	27.9	20.4	5.8	6.2	17.6	29.9
**L7**	72.1	53.7	41.9	35.0	28.6	20.1	4.6	5.8	14.8	26.6
**L8**	71.1	58.1	53.4	45.0	33.0	23.5	6.7	6.6	19.7	33.3
**L9**	58.9	44.7	40.0	33.9	29.9	20.8	6.2	11.5	14.8	27.3
**L10**	101.5	63.6	90.9	68.9	46.6	35.6	9.2	8.7	30.7	46.1
**MeanL**	**69.0**	**53.1**	**49.6**	**43.7**	**31.7**	**23.5**	**5.5**	**6.6**	**19.1**	**33.3**
**Mean**	**64.4**	**50.3**	**46.1**	**41.1**	**30.7**	**23.0**	**6.5**	**7.2**	**25.4**	**41.9**

1.2 Figures

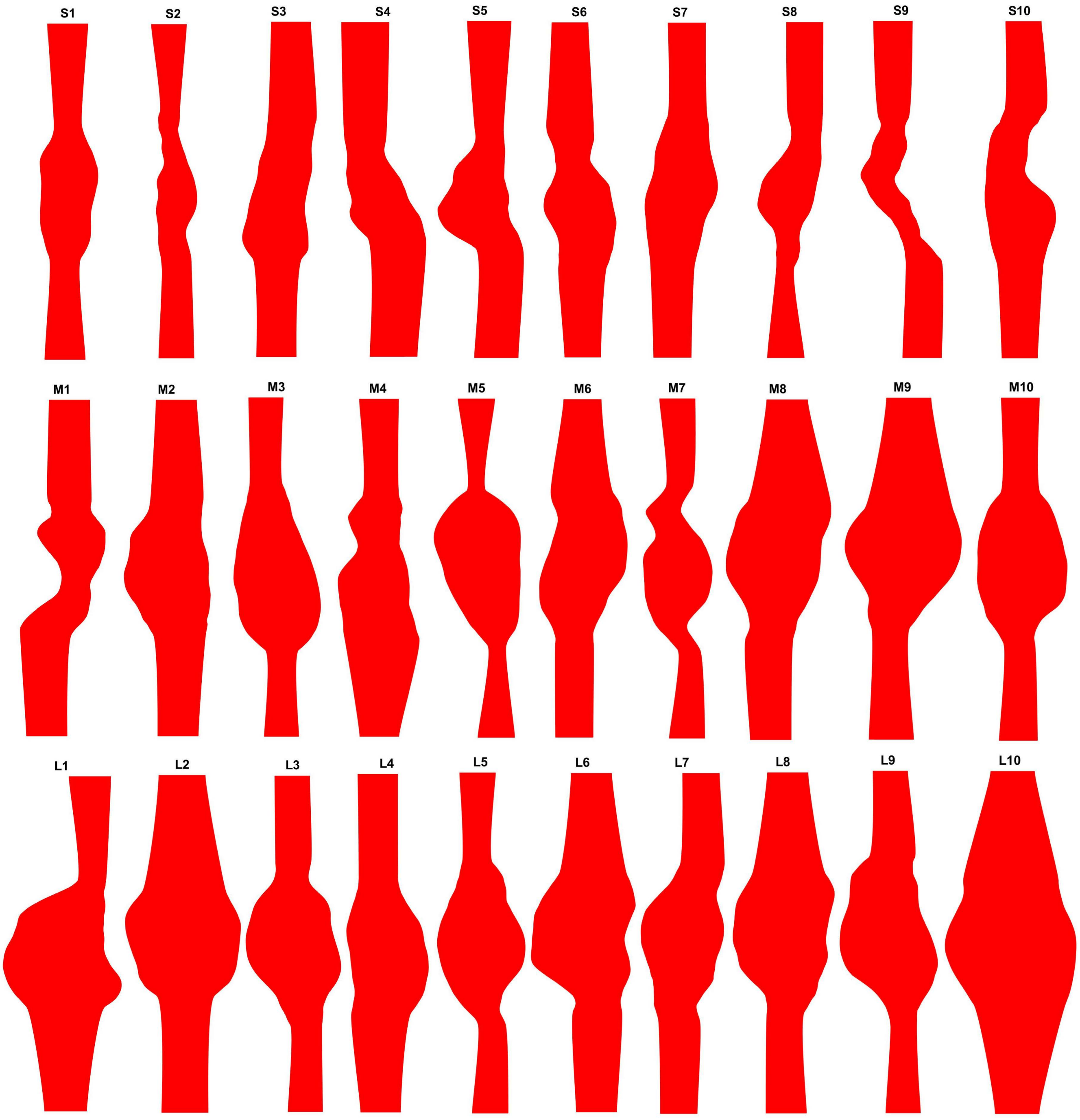

Figure S1. 3D+t US-based diastolic lumen surfaces meshes for all 30 patients included, demonstrating the large variety in AAA geometry

2 MESH CONVERGENCE STUDY

A mesh convergence study was executed to ensure the use of proper mesh size. This study was executed with an older framework, in which CT data was used as input for the FSI framework instead of 3D+t US data. However, the main computational features have remained the same for this study.

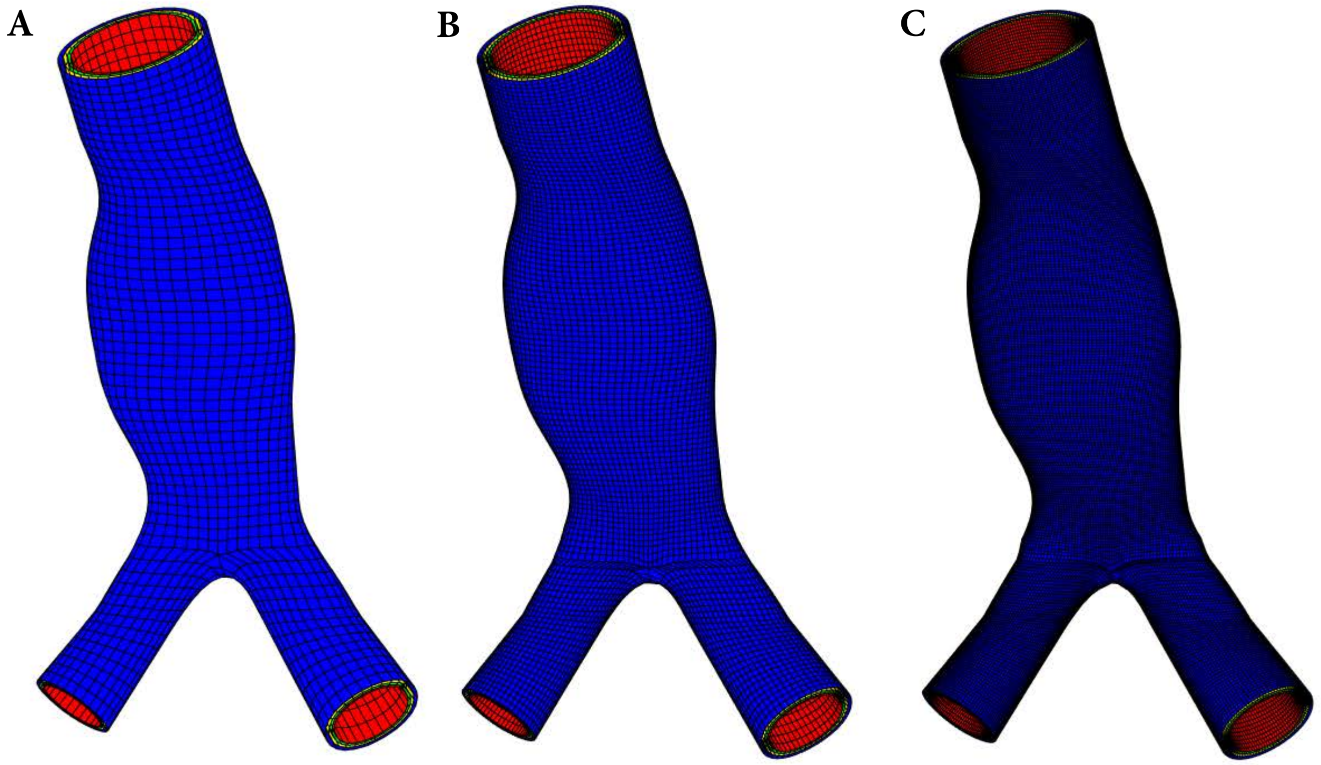

Figure S2. Surface meshes with a mesh size of (A) 1.6 mm, (B) 0.8 mm and (C) 0.4 mm

2.1 Structural domain

The effect of the mesh size on the accuracy of the structural model was investigated by altering the mesh size of the surface mesh and the number of layers separately.

To adjust the mesh size of the surface mesh, additional contours and points were added to the mesh. Special care was taken to preserve the mesh shape when adjusting the mesh size. Therefore, the mesh size was adjusted after the mesh was smoothed. For 3 patients, surface meshes with mesh sizes of 1.6, 0.8, and 0.4 mm were obtained, as shown in Figure S2. Using these surface meshes, quadratic hexahedral volume meshes with 3 wall layers were created. The models were executed without pre-stress following the method outlined in Section 2.2, except for the boundary conditions. For this study, the inner edges of the inlet and outlets were fixed in all directions. In 16 subsequent load steps, the pressure curve shown in Figure S3 was applied to each of the models.

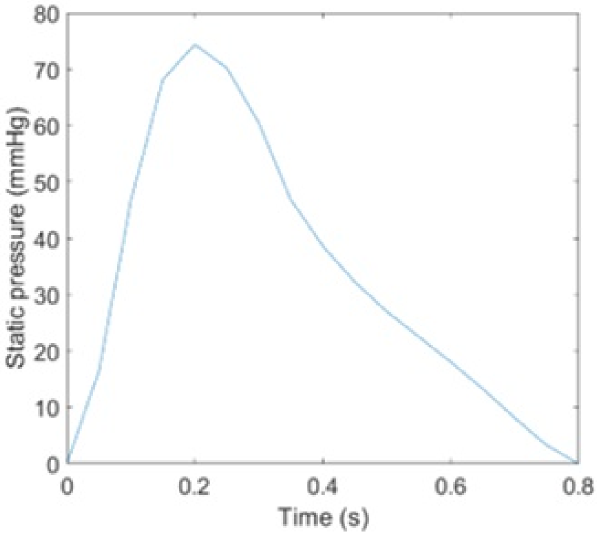

Figure S3. Pressure curve applied to the structural models

Figure S4A shows a summary of the statistics of the different models. Halving the mesh size causes an increase in the number of elements by approximately a factor of 4. The computation time increases with a factor of approximately 5.

The average Von Mises stress is plotted against the time for each model (Figure S4B). The different

A

Model Name	Mesh size	Number of elements	Run time
AAA-1	1.6 mm	7.443	31min
	0.8 mm	29.232	2h 26min
	0.4 mm	115.620	12h 23min
AAA-2	1.6 mm	11.598	49 min
	0.8 mm	45.708	4h 7 min
	0.4 mm	180.120	18h 59min
AAA-3	1.6 mm	14.745	52 min
	0.8 mm	57.510	5h 21min
	0.4 mm	229.536	29h 24min

B

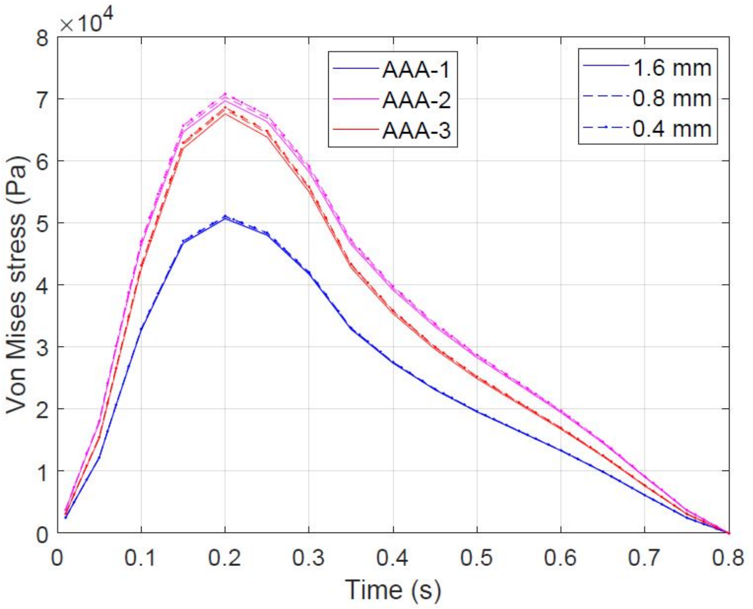

C

	1.6 vs 0.8 mm	0.8 vs 0.4 mm	1.6 vs 0.4 mm
AAA-1	0.55%	0.30%	0.85%
AAA-2	0.87%	0.71%	1.59%
AAA-3	1.02%	0.50%	1.53%

Figure S4. Results of the models with different mesh sizes. (A) Summary of the statistics of the different models. (B) Average Von Mises stress plotted against the time for all models. The different geometries are indicated with different colors, whereas the different mesh sizes are indicated with different markers. (C) Percentual difference of the Von Mises stress for the different models at peak systole

A

Model Name	Number of layers	Number of elements	Run time
AAA-1	1	9.744	52min
	2	19.488	1h 22min
	4	38.976	4h 43min
AAA-2	1	15.236	1h 12min
	2	30.472	2h 21min
	4	60.944	7h 37min
AAA-3	1	19.170	1h 40min
	2	38.340	3h 2min
	4	76.680	9h 50min

B

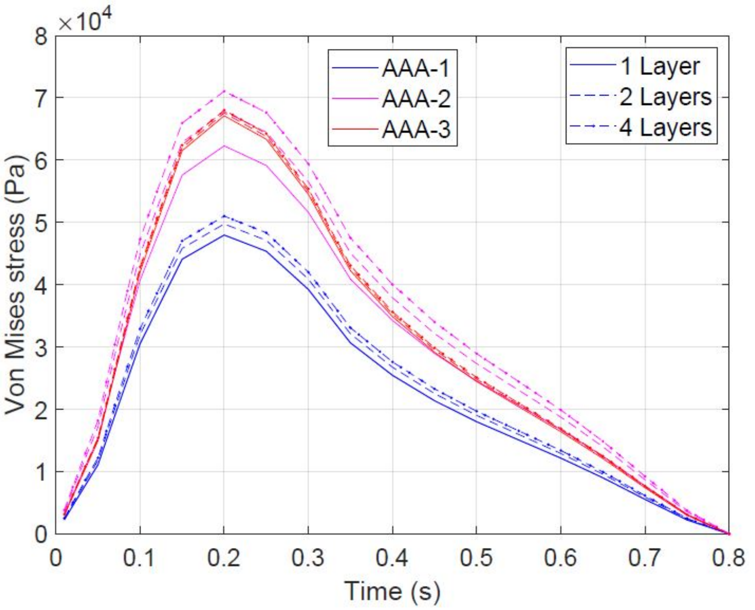

C

	1 layer vs 2 layers	2 layers vs 4 layers	1 layer vs 4 layers
AAA-1	3.71%	2.53%	6.34%
AAA-2	8.85%	4.79%	14.07%
AAA-3	0.68%	0.66%	1.35%

Figure S5. Results of the models with different number of layers. (A) Summary of the statistics of the different models. (B) Average Von Mises stress plotted against the time for all models. The different geometries are indicated with different colors, whereas the different number of wall layers are indicated with different markers. (C) Percentual difference of the Von Mises stress for the different models at peak systole

patients are indicated with different colors, whereas the different mesh sizes are indicated with different markers. For all patients, convergence in results is visible. This convergence is quantified by comparing the

average Von Mises stress of the different models at peak systole (t = 0.2 s) as shown in Figure S4C. The increase in accuracy of the 0.4 mm mesh does not outweigh the increase in computation time. Next to the increase in accuracy, the 0.8 mm mesh provides higher stability compared to the 1.6 mm mesh, since the height-length ratio of the hexahedral elements decreases. Consequently, this convergence study showed that a mesh size of 0.8 mm provides the best balance between accuracy and computation time.

In a second convergence study, the effect of the number of wall layers on the accuracy of the structural model was examined. For each patient, models with 1, 2 and 4 wall layers were obtained and executed. The mesh size of the surface mesh was set to 0.8 mm, as found in the previous convergence study. No growth ratio was applied to the layers, indicating that the thickness of each layer is equal.

Doubling the number of layers induces a doubling of the number of elements, as shown in Figure S5A. This figure also shows that the computation time increases non-linearly.

The average Von Mises stress is plotted against the time in Figure S5B. Again, the different patients are indicated with different colors and the different number of wall layers are indicated by different markers. The convergence in the solution is quantified by comparing the average Von Mises stress of the different models at peak systole (t = 0.2 s) and shown in Figure S5C. Clear convergence is seen, especially for patients AAA-1 and AAA-2. The increase in accuracy of the 4-layer mesh does not outweigh the increase in computation time. Therefore, this convergence study showed that a mesh containing 2 wall layers provides the best balance between accuracy and computation time.

2.2 Fluid domain

The volume mesh size of the fluid mesh was altered to investigated the effect of the mesh size on the accuracy of the fluid model. Volume mesh sizes of 2, 1 and 0.5 mm were used to obtain the different meshes (Figure S6). This was done for 3 patients, resulting in a total of 9 models in this convergence study.

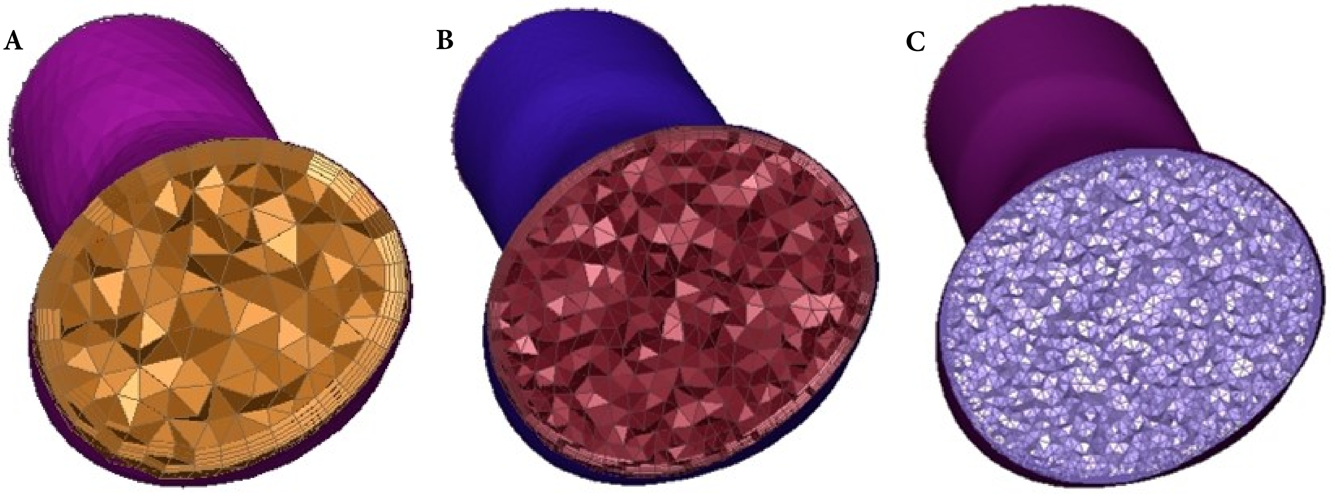

Figure S6. Lumen volume meshes using a mesh size of (A) 2, (B) 1 or (C) 0.5 mm

All models were executed following the method outlined in Section 2.3, except for the inlet velocity profile. Instead of using the inlet flow shown in Figure 1 to determine the inlet velocity, the velocity profile as shown in Figure S7A was prescribed, which resulted in a lower centerline velocity. Since the fluid and solid domain were simulated separately for the convergence studies, the AAA wall was rigid (no compliance of the AAA wall). A heart rate of 75 beats per minute was prescribed and a total of 5 cardiac

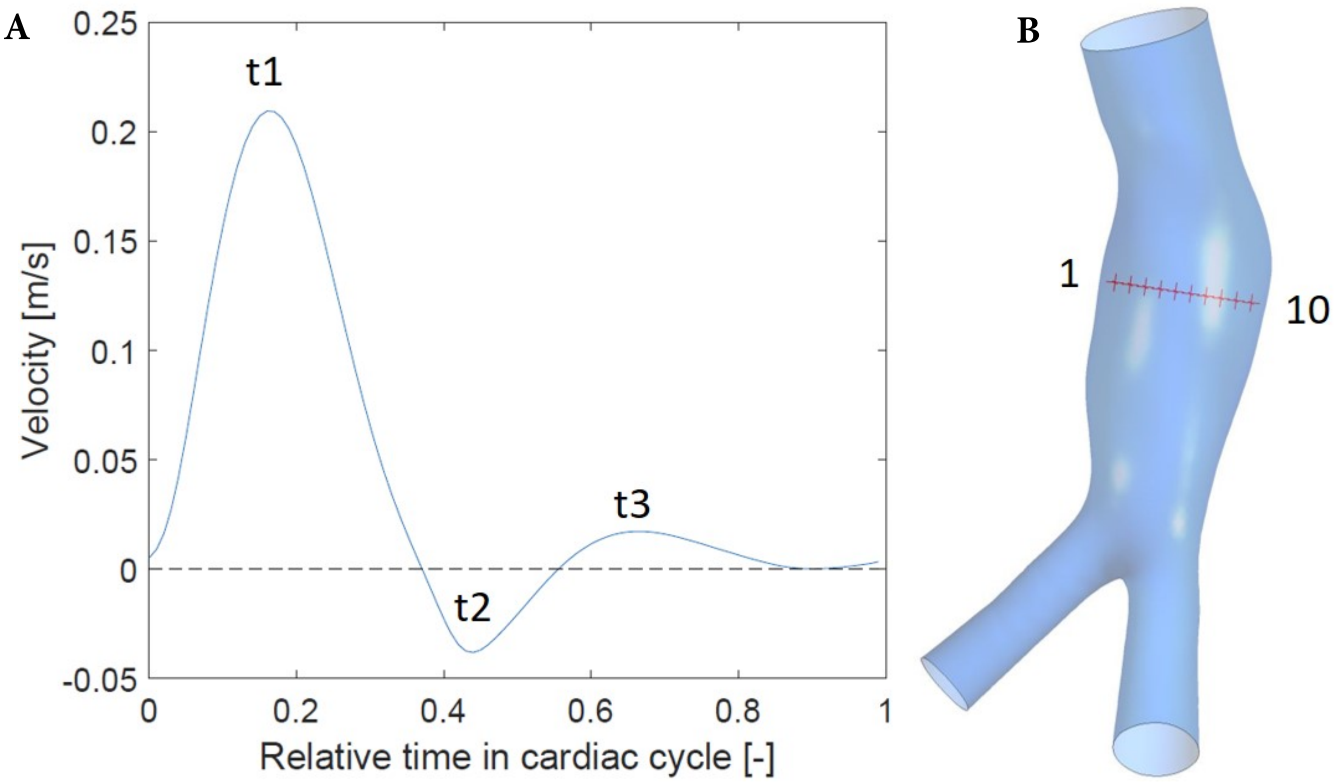

Figure S7. The velocity results were extracted at 3 points in time, as indicated in (A) on 10 cross-sectional points as shown in (B)

cycles were simulated. The velocity results of the last cardiac cycle were evaluated on 3 different instants in time, (Figure S7A) on 10 cross-sectional points (Figure S7B) in the aneurysm region. The cross-sectional points for each geometry were obtained in Matlab, using the surface mesh. For each geometry, the same 10 points were used to evaluate the results of the models with different mesh sizes.

A summary of the statistics of the different models is shown in Figure S8A. Halving the mesh size causes an increase in the number of elements by approximately a factor of 5. The computation time increases exponentially.

The results of the convergence study are shown in Figure S8B-D. For each patient, the velocity in each of the cross-sectional points is plotted for each time point. The different time points are indicated in blue, magenta and red, whereas the different mesh sizes are indicated with different markers.

For most points, the differences in velocities between the 2 mm and 1 mm mesh are bigger than the difference in velocities between the 1 mm and 0.5 mm mesh, which indicates convergence of the solution. A few exceptions are seen at points close to the boundaries of the mesh. The increase in accuracy of the 0.5 mm mesh does not outweigh the increase in computation time. Therefore, this convergence study showed that a mesh size of 1 mm provides the best balance between accuracy and computation time.

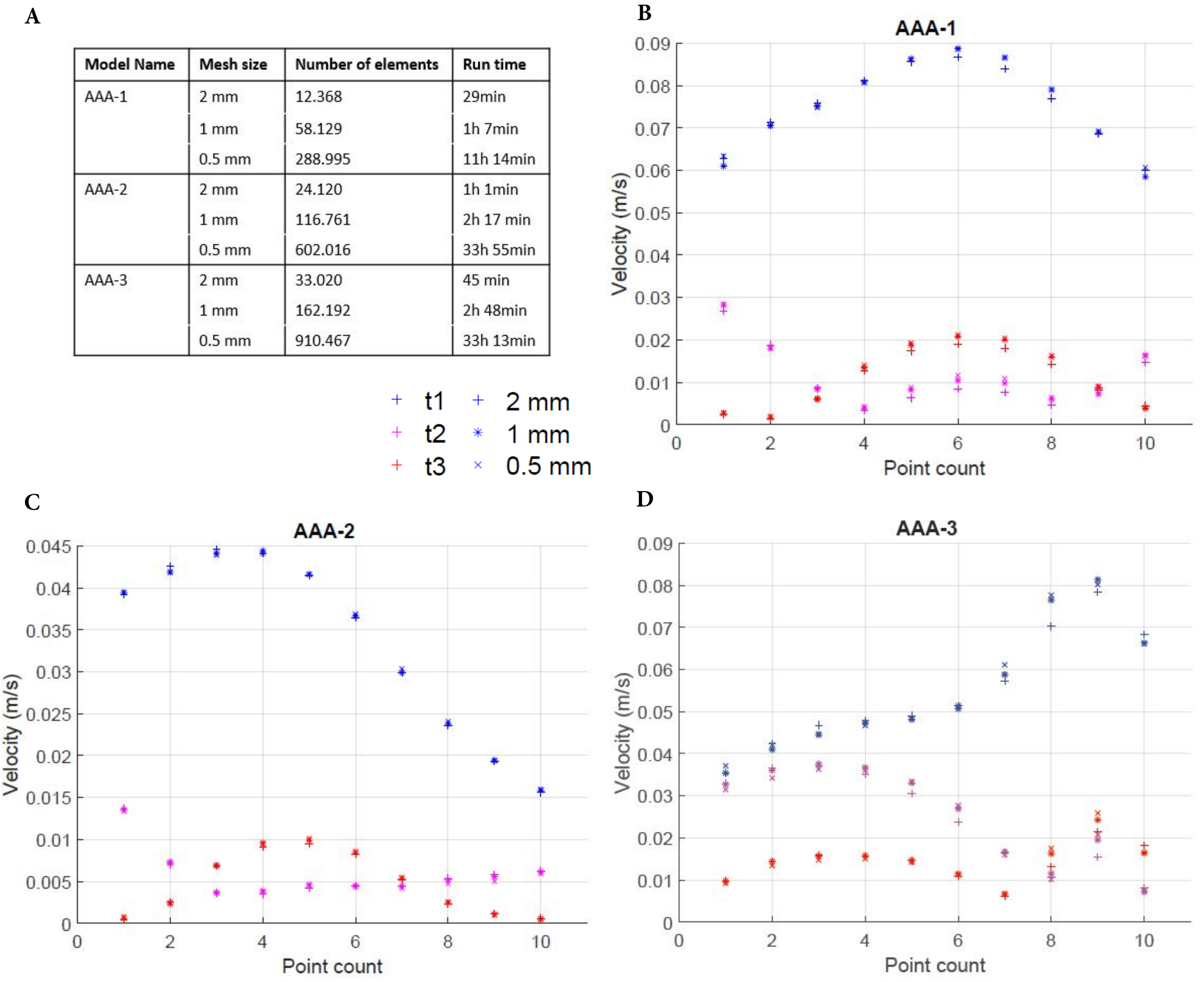

Model Name	Mesh size	Number of elements	Run time
AAA-1	2 mm	12.368	29min
	1 mm	58.129	1h 7min
	0.5 mm	288.995	11h 14min
AAA-2	2 mm	24.120	1h 1min
	1 mm	116.761	2h 17 min
	0.5 mm	602.016	33h 55min
AAA-3	2 mm	33.020	45 min
	1 mm	162.192	2h 48min
	0.5 mm	910.467	33h 13min

Figure S8. (A) Summary of the statistics of the different models. (B-D) Velocity at 10 cross-sectional points for patients (B) AAA-1, (C) AAA-2 and (D) AAA-3. The different time points are indicated in different colors, whereas the different mesh sizes are indicated with different markers
